# Boosting electrocatalytic CO_2_–to–ethanol production via asymmetric C–C coupling

**DOI:** 10.1038/s41467-022-31427-9

**Published:** 2022-06-29

**Authors:** Pengtang Wang, Hao Yang, Cheng Tang, Yu Wu, Yao Zheng, Tao Cheng, Kenneth Davey, Xiaoqing Huang, Shi-Zhang Qiao

**Affiliations:** 1 School of Chemical Engineering and Advanced Materials, The University of Adelaide, Adelaide, SA 5005 Australia; 2 State Key Laboratory of Physical Chemistry of Solid Surfaces, College of Chemistry and Chemical Engineering, Xiamen University, Xiamen, 361005 China; 3 Institute of Functional Nano & Soft Materials (FUNSOM), Jiangsu Key Laboratory for Carbon-Based Functional Materials & Devices, Joint International Research Laboratory of Carbon-Based Functional Materials and Devices, Soochow University, Suzhou, 215123 China

**Keywords:** Electrocatalysis, Energy, Electrocatalysis

## Abstract

Electroreduction of carbon dioxide (CO_2_) into multicarbon products provides possibility of large-scale chemicals production and is therefore of significant research and commercial interest. However, the production efficiency for ethanol (EtOH), a significant chemical feedstock, is impractically low because of limited selectivity, especially under high current operation. Here we report a new silver–modified copper–oxide catalyst (dCu_2_O/Ag_2.3%_) that exhibits a significant Faradaic efficiency of 40.8% and energy efficiency of 22.3% for boosted EtOH production. Importantly, it achieves CO_2_–to–ethanol conversion under high current operation with partial current density of 326.4 mA cm^−2^ at −0.87 V vs reversible hydrogen electrode to rank highly significantly amongst reported Cu–based catalysts. Based on in situ spectra studies we show that significantly boosted production results from tailored introduction of Ag to optimize the coordinated number and oxide state of surface Cu sites, in which the ^*^CO adsorption is steered as both atop and bridge configuration to trigger asymmetric C–C coupling for stablization of EtOH intermediates.

## Abstract (web summary)

It is of high interest to convert CO_2_ into valuable ethanol product. Here the authors demonstrate the asymmetric C-C coupling triggered on Ag-modified oxide-derived Cu sites can accelerate and steer the reaction pathway for ethanol production with high faradaic efficiency and current density.

## Introduction

Electroreduction of carbon dioxide (CO_2_) to high-value chemicals and fuels is seen to be practically promising for the utilization of renewable electricity and mitigation of CO_2_ emissions, which has emerged as a frontier in energy conversion and carbon neutrality^1–3^. During the CO_2_ reduction reaction (CO_2_RR), the applied electrical energy is converted to stored chemical energy via reorganizing the molecular bonds in CO_2_ and water to generate products with one (C_1_), or two or more (C_2+_) carbon atoms, under the effect of catalysts^4–6^. Most metal catalysts such as gold (Au), silver (Ag), tin (Sn), and lead (Pb) generate a mix of C_1_ products^7,8^, whilst only Cu-based catalysts transform CO_2_ toward C_2+_ products via coupling the adsorbed ^*^CO intermediates^9–11^. Amongst the various C_2+_ products formed on Cu catalysts, EtOH is important as the liquid fuel because of its wide application and high-energy-density and because it provides the possibility of long-term, large-scale and seasonal energy storage, and convenient transport^3,12^. The production of EtOH with high current density and Faradaic efficiency (FE) via Cu-based catalysts is significant to advancing CO_2_RR as a renewable chemical feedstock^2,3,13^. During CO_2_RR, EtOH and ethylene (C_2_H_4_) are both 12-electron reduced products, and share the initial intermediates ^*^HCCOH. Given the more saturated structure of EtOH compared with that for C_2_H_4_, the next-intermediates for EtOH are more difficult to stabilize on a pure Cu surface compared with C_2_H_4_. The production of EtOH via C–O bond-reserving of ^*^HCCOH will therefore have chemical difficulty in competing with C_2_H_4_ generation. This typically results in the EtOH production in the range of 2–3 times less than that for C_2_H_4_ on Cu-based catalyst^3,11–14^.

To boost EtOH production, research interest has concentrated on optimal re-design of Cu-based catalysts^5,6,15–22^. Strategies including, control of morphology and facet^16,17^, vacancy steering^18^, dopant and modification engineering^19,20^, and defects control^21,22^ have been reported. Among these, the modification of Cu with other CO_2-_–active metals to form Cu-based bimetallic is reported as practically attractive^15,23–28^. For example, Jaramillo et al. reported Cu–Au bimetallic catalysts with boosted selectivity for CO_2_RR to EtOH, and a synergistic catalytic pathway with CO–tandem mechanism was proposed that CO was first generated on Au, desorbed and migrated to near Cu active sites where C–C coupling to EtOH was conducted^24^. Zheng et al. reported a boosted EtOH selectivity in a Cu_3_Ag_1_ bimetallic catalyst with electron-deficient Cu sites via promoting adsorption of key intermediates^25^. Clark et al. hypothesized that the boosted EtOH selectivity results from Ag-induced strain effects of Cu surfaces that modulate EtOH production and suppresses the hydrogen evolution reaction (HER)^26^. Despite significant progress, the advances of reported bimetallic catalysts for FE of EtOH (FE_EtOH_) remain limited, especially the production and output efficiency for EtOH is far from the current target for practical application^29–38^ (i.e., partial current density > 300 mA cm^−2^ and half-cell cathodic energy efficiencies (EE_HC_) > 20%). In addition, the key impact of modified components on intrinsic kinetics of reported Cu-based bimetallic catalysts for CO_2_RR at high conversion rates is unclear, which significantly hinders understanding of the mechanism and catalyst design^3,14^. Taken together, there is a need therefore for more efficient catalysts and an improved understanding of the mechanism for CO_2_RR to practically boost EtOH under commercial current densities.

Here, through modifying Ag onto cubic Cu_2_O and activating under CO_2_RR, we investigated derived CuAg bimetallics (dCu_2_O/Ag) with controlled morphology, phase, and composition for CO_2_RR at high current operation. In contrast to the Cu_2_O and Au-modified Cu_2_O derivatives which favor the conversion of CO_2_ to C_2_H_4_ and CO, respectively, the optimal dCu_2_O/Ag_2.3%_ exhibited an asymmetric C–C coupling to stabilize reaction intermediates for boosted EtOH production under high current density. The as-obtained dCu_2_O/Ag_2.3%_ exhibits FE and EE_HC_ for EtOH of, 40.8% and 22.4%, respectively. We show in a direct comparison with reported catalysts that it has the greatest reported partial EtOH current density with 326.4 mA cm^−2^. In situ studies confirm that the redispersion of Ag into Cu significantly optimizes the coordinated number and oxide state of Cu. In this way, the ^*^CO binding strength is altered to form a blended adsorption configuration, that triggers asymmetric C–C coupling for stabilization of EtOH intermediates, and results in boosted EtOH production. This work constructs an efficient catalyst for CO_2_RR with high EtOH selectivity at commercially relevant current densities, and provides guidance for designing catalysts with tailored selectivity in multi-electron reactions.

## Results

### Catalyst preparation and characterization

**Fig. 1:**
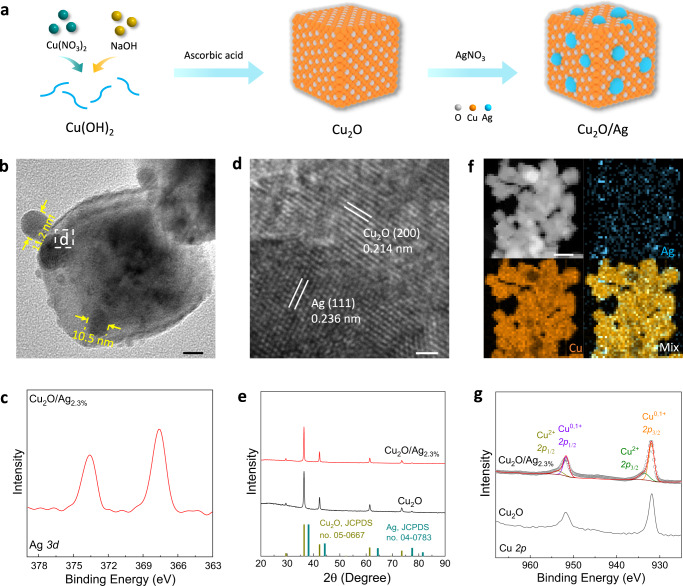
Structural characterization of Cu_2_O/Ag NCs. **a** Schematic for preparation of Cu_2_O/Ag NCs. **b** TEM image, **c** Ag *3d* XPS curve, **d** HRTEM images and **f** EDS elemental mapping images of Cu_2_O/Ag_2.3%_ NCs. **e** XRD patterns and **g** Cu *2p* XPS curves for Cu_2_O and Cu_2_O/Ag_2.3%_ NCs. Scale bars, 10 nm in (**b**), 1 nm in (**d**), and 100 nm in (**f**). White-color, orange and azure spheres in the model represent O, Cu, and Ag atoms, respectively.

Pristine Ag-modified Cu_2_O nanocubes (Cu_2_O/Ag NCs) were prepared by a one-pot seed-medium method in which Cu_2_O NCs were achieved via reduction of Cu(OH)_2_ at room temperature (RT, 25 °C) with ascorbic acid (AA) as a reducing agent, followed by the addition of AgNO_3_. Because of the appropriate lattice spacing match, the added AgNO_3_ rapidly nucleates and grows due to the effect of AA to deposit ‘small’ Ag nanoparticles (Ag NPs) on Cu_2_O NCs surface (Fig. 1a). Transmission electron microscopy (TEM) images reveal that the Cu_2_O/Ag NCs exhibit heterostructure that Ag NPs sporadically adorn the Cu_2_O surface (Fig. 1b and Supplementary Fig. 1a), in comparison to Cu_2_O NCs with cubic morphology and a side length of ~45 nm (Supplementary Fig. 2). X-ray photoelectron spectroscopy (XPS) and scanning electron microscopy energy-dispersive X-ray spectroscopy (SEM-EDS) confirm that the Ag NPs in Cu_2_O/Ag NCs are metallic, with the content controlled to 2.3% (Fig. 1c and Supplementary Fig. 1b). High-resolution TEM (HRTEM) image highlights the interplanar spacing of the lattice fringes for Cu_2_O NCs and Ag NPs regions to be 0.214 nm and 0.236 nm in Cu_2_O/Ag_2.3%_ NCs. This finding is consistent with the Cu_2_O (200) plane and Ag (111) plane, respectively (Fig. 1d). Powder X-ray diffraction (XRD) was carried out to confirm the phase of Cu_2_O/Ag NCs. It was found that Cu_2_O/Ag NCs exhibit the same peak as for Cu_2_O NCs which is attributed to the low Ag NPs content (Fig. 1e). In addition, the EDX mapping images for Cu_2_O/Ag_2.3%_ NCs also display an apparent element separation between Ag NPs and Cu_2_O NCs, (Fig. 1f). XPS assesses the surface properties of catalysts. The peaks at 951.8 and 931.8 eV for Cu_2_O NCs are ascribed to, Cu *2p*_1/2_ and *2p*_3/2_, respectively, confirming the presence of Cu(I) in Cu_2_O NCs (Fig. 1g)^39^. Following Ag modification, two shoulder peaks for Cu(II) were apparent in the XPS spectra, demonstrating that electrons transfer from Cu_2_O to Ag. This finding is validated via the Auger electron spectroscopy (AES) for Cu LMM in which the peaks for Cu_2_O/Ag_2.3%_ NCs downshifts to a lower kinetic energy compared with those for Cu_2_O NCs (Supplementary Fig. 3). These findings indicate that an Ag/Cu_2_O heterostructure with altered electron structure for Cu_2_O was reached with Cu_2_O/Ag_2.3%_ NCs.

### Catalyst evolution under CO_2_RR

Given the reported evolution of Cu-based catalysts under high current CO_2_RR^36^, activation and in situ characterization was therefore conducted for Cu_2_O/Ag_2.3%_ NCs to determine the actual state of the catalyst during CO_2_RR. A flow cell with gas diffusion electrode (GDE) was especially designed as a reactor for a high current test (Supplementary Fig. 4). As a basis for a detailed comparison, Cu_2_O NCs and Au-modified Cu_2_O NCs (Cu_2_O/Au_2.3%_ NCs) with similar morphology, composition, and structure to Cu_2_O/Ag_2.3%_ NCs were synthesized and assessed (Supplementary Fig. 5). The catalysts were deposited onto the GDE via spray-coating of the configured ink (details in Supplementary Methods). Activation was controlled by electroreduction of the parent material under CO_2_RR at a current density of 200 mA cm^−2^ in 1 M KOH for 30 min. The derived Cu-based catalysts obtained following activation supported on the GDE, denoted as dCu_2_O, dCu_2_O/Ag, and dCu_2_O/Au, were subjected to additional characterization.

**Fig. 2:**
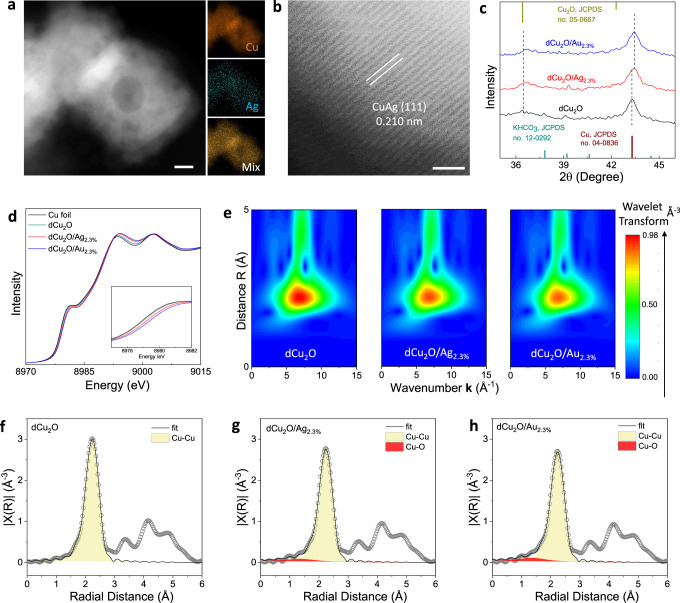
Phase and coordination environment for dCu_2_O/Ag catalysts. **a** HAADF–STEM image with EDS elemental mappings and **b** high resolution STEM image of dCu_2_O/Ag_2.3%._ **c** Enlarged XRD patterns for dCu_2_O, dCu_2_O/Ag_2.3%_ and dCu_2_O/Au_2.3%_. **d** In situ XANES spectra and **e** Wavelet transform images of EXAFS data with optimized Morlet parameter (i.e., *κ* = 5, *σ* = 1) at Cu K-edge for commercial Cu-foil, dCu_2_O, dCu_2_O/Ag_2.3%_ and dCu_2_O/Au_2.3%_. Fourier transform curves of in situ EXAFS data and corresponding fitted results (first coordination shell) for **f** dCu_2_O, **g** dCu_2_O/Ag_2.3%_ and **h** dCu_2_O/Au_2.3%_. Scale bars, 10 nm in (**a**) and 1 nm in (**b**).

High-angle annular dark-field scanning TEM (HAADF STEM) and SEM images confirm that following activation the original cubic morphology and surface deposited NPs are visually less pronounced, and that instead, ragged surface and hollow structures formed in dCu_2_O/Ag_2.3%_ and dCu_2_O/Au_2.3%_ (Fig. 2a and Supplementary Figs. 6 and 7a). Concomitantly, the original surface phase separation between deposited metal and Cu is lost following activation as is confirmed in EDS mapping images (Fig. 2a, Supplementary Fig. 7b). The lattice space for dCu_2_O/Ag_2.3%_ was altered to 0.210 nm following activation, a value near to the Cu (111) facet (Fig. 2b). Ex situ XRD patterns reveal that the diffraction peaks for Cu_2_O for all catalysts are decreased significantly whilst the diffraction peaks for Cu became dominant. This finding demonstrates that all catalysts are transformed to mainly metallic Cu following activation (Supplementary Fig. 8). Compared with dCu_2_O, the XRD peaks of Cu (111) for dCu_2_O/Ag_2.3%_ and dCu_2_O/Au_2.3%_ exhibit a meaningful, slight shift to a higher degree to underscore that the original Cu_2_O/metal heterostructures are evolved into the bimetallic alloy following activation (Fig. 2c).

To gain insight into the changed valence states and coordination environment of Cu following activation, *operando* X-ray absorption spectra (XAS) of Cu K-edge was recorded under activation conditions. The X-ray absorption near-edge structure (XANES) spectra show that the edge features for dCu_2_O, dCu_2_O/Ag_2.3%_, and dCu_2_O/Au_2.3%_ are closed to those for the reference commercial Cu-foil. This finding confirms that the valence state for Cu for these catalysts decrease following activation to lie between 0 and +1, in following ordered sequence, dCu_2_O < dCu_2_O/Ag_2.3%_ < dCu_2_O/Au_2.3%_ (Fig. 2d). The wavelet transform analysis confirms that the Cu–Cu region in all activated catalysts are located at ~6.7 Å^−1^, suggesting that the alloyed CuAg and CuAu do not result in significant change in Cu–Cu bond length compared with the oxide-derived Cu (Fig. 2e). The corresponding Fourier transform curves (from extended X-ray absorption fine structure (EXAFS) spectra) and fitted results of the first coordination shell for these activated catalysts show that the Cu–Cu coordination located at ~2.23 Å is the dominant structure in each sample, whilst there remains another Cu–O coordination peak at 1.35 Å for dCu_2_O/Ag_2.3%_ and dCu_2_O/Au_2.3%._ This finding confirms that a significantly small fraction of Cu(I) remained for dCu_2_O/Ag_2.3%_ and dCu_2_O/Au_2.3%_ following activation (Fig. 2f–g and Supplementary Table 1). The coordination number for Cu−Cu in these catalysts was determined to equal, respectively, 11.4, 10.5, and 10.2 for dCu_2_O, dCu_2_O/Ag_2.3%_, and dCu_2_O/Au_2.3%_ (Supplementary Fig. 9). Except for variation in Cu, the XPS for Ag *3d* for dCu_2_O/Ag_2.3%_ shows that the binding energy for Ag *3d*_5/2_ shifts to a high level following activation. This evidences that the activation of alloying Ag with Cu induces electron transfer (Supplementary Fig. 10). Based on the foregoing, it is concluded that under electroreduction with highly significant structural rearrangement, the pristine metal-modified Cu_2_O NCs evolves to Cu-based bimetallics in a regulated valence and coordination environment.

### CO_2_RR performance

**Fig. 3:**
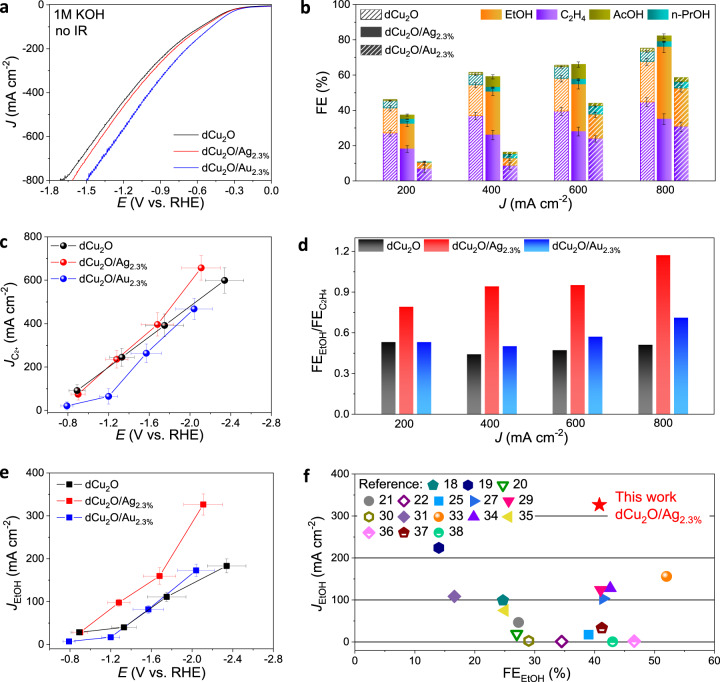
CO_2_RR performance for dCu_2_O, dCu_2_O/Au_2.3%_ and dCu_2_O/Ag_2.3%_. **a** Linear sweep voltammetry curves toward CO_2_RR for dCu_2_O, dCu_2_O/Au_2.3%_ and dCu_2_O/Ag_2.3%_. **b** FE value of C_2+_ products for dCu_2_O, dCu_2_O/Au_2.3%_ and dCu_2_O/Ag_2.3%_ under selected current density. **c** Partial C_2+_ current density and **e** C_2+_ formation *vs* potential referred to reversible hydrogen electrode (RHE) on dCu_2_O, dCu_2_O/Au_2.3%_, and dCu_2_O/Ag_2.3%_. **d** Ratio of FE_EtOH_ to FE_C2H4_ on dCu_2_O, dCu_2_O/Au_2.3%_, and dCu_2_O/Ag_2.3%_ under selected current density. **f** EtOH partial current density *vs* FE_EtOH_ for reported Cu-based catalysts. Error bars correspond to the standard deviation of three independent measurements.

CO_2_RR performance for the activated catalysts was directly evaluated via electrolyzing at specified currents (Supplementary Fig. 11). Figure 3a shows the linear sweep voltammetry curves for dCu_2_O, dCu_2_O/Ag_2.3%_ and dCu_2_O/Au_2.3%_. It is seen in the figure that the current density for CO_2_RR of dCu_2_O/Ag_2.3%_ and dCu_2_O/Au_2.3%_ are significantly increased, directly evidencing that modification boosted activity for CO_2_RR. The FEs were computed for liquid and gaseous product in the applied current range 200–800 mA in 1 M KOH by nuclear magnetic resonance (NMR, Supplementary Fig. 12) and gas chromatography (GC) (Supplementary Fig. 13) respectively. Figure 3b presents the FEs of C_2+_ products (FEs_C2+_) for these catalysts under different current. C_2_H_4_ and EtOH are the major C_2+_ products, plus minor acetate and n–propanol. With the applied current increased, all catalysts exhibited increased FEs_C2+_ and a decreased FEs of CO (FEs_CO_). Compared with the one–up FE_C2+_ for dCu_2_O from 200 to 400 mA, dCu_2_O/Ag_2.3%_ exhibits greater FE_C2+_ at significant current >  600 mA. Importantly, the total FE_C2+_ for dCu_2_O/Ag_2.3%_ is up to 82.1% at a current density 800 mA cm^−2^, to exhibit the greatest partial C_2+_ current density of 656.8 mA cm^−2^ and formation rate of 2042.2 μmol h^−1^ cm^−2^ at −2.11 V with reference to the reversible hydrogen electrode (V_RHE_, no *iR* correction, Fig. 3c and Supplementary Fig. 14a). In contrast, the FE_C2+_ for dCu_2_O/Au_2.3%_ is significantly less than that for dCu_2_O/Ag_2.3%_ at all currents, confirming that the Au modification resulted in directly boosting only targeted CO generation, but not C–C coupling.

We analyzed the ratio of FE_EtOH_/FE_C2H4_ in C_2+_ products at high current density on these catalysts. It is interesting that dCu_2_O/Ag_2.3%_ reaches the FE_EtOH_/FE_C2H4_ ratio 1.17 at an applied current 800 mA, which is significantly greater than that for dCu_2_O (0.51) and dCu_2_O/Au_2.3%_ of 0.71. This finding confirms that the Ag modification significantly inhibits C–O bond-breaking and stabilizes intermediates for EtOH vs C_2_H_4_ (Fig. 3d). Relying on the boosted FE_EtOH_ of 40.8 % at a high current density (800 mA cm^−2^), the dCu_2_O/Ag_2.3%_ exhibits the greatest partial EtOH current density of 326.4 mA cm^−2^ at −2.11 V_RHE_ (no *iR* correction, −0.89 V_RHE_ with 85% *iR* correction, Supplementary Figs. 15 and 16). Importantly, this is 1.78 and 1.89 times greater than that for dCu_2_O and dCu_2_O/Au_2.3%_, respectively (Fig. 3e). The EtOH formation for dCu_2_O/Ag_2.3%_ could reach 1014.9 μmol h^−1^ cm^−2^ with current densities of 800 mA cm^−2^ at −2.11 V_RHE_ (without *iR* correction) (Supplementary Fig. 14b). Such highly significant performances for EtOH production on dCu_2_O/Ag_2.3%_ were compared directly with reported catalysts (Fig. 3f, Supplementary Table 2). It is apparent that the dCu_2_O/Ag_2.3%_ exhibits the greatest reported partial EtOH current density amongst these, and represents the ‘best’ production for electroreduction of CO_2_ to EtOH. In addition, the EE_HC_ for EtOH on dCu_2_O/Ag_2.3%_ reaches 22.4% following *iR* correction under similar conditions, a value greater than that for most catalysts (Supplementary Fig. 16d). In addition, the electrochemically active surface area (ECSA) for dCu_2_O/Ag_2.3%_ was computed via different methods and compared, together with mass normalized current density for EtOH (J_ECSA_(EtOH) and J_mass_(EtOH)) with independently reported studies (Supplementary Figs. 17 and 18 and Supplementary Table 3). The dCu_2_O/Ag_2.3%_ exhibited superior J_ECSA_(EtOH) and J_mass_(EtOH) compared with reported catalysts, confirming that the improved EtOH current density for dCu_2_O/Ag_2.3%_ is because of the intrinsic Ag-modified oxide-derived Cu sites and not the changed ECSA and mass loading of the catalyst itself.

**Fig. 4:**
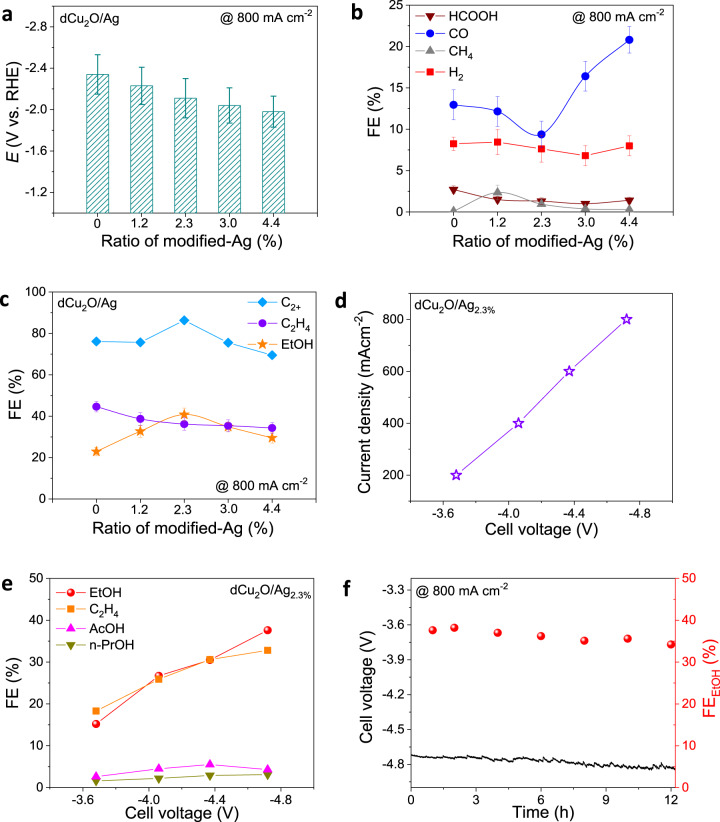
CO_2_RR performance for dCu_2_O/Ag with modified Ag. Comparison of **a** applied potentials, **b** FEs for C_1_ and H_2_ product, **c** FEs for C_2_H_4_ and EtOH and total C_2+_ product on dCu_2_O/Ag with modified Ag at current density 800 mA cm^−2^. **d** Total current density and **e** FEs for C_2+_ product for CO_2_RR on dCu_2_O/Ag_2.3%_ at selected cell voltage under MEA measurement. **f** Stability test for dCu_2_O/Ag_2.3%_ at the current density 800 mA cm^−2^ in MEA. Error bars correspond to the standard deviation of three independent measurements.

Based on these findings therefore of highly significant FE_EtOH_ with high current density from dCu_2_O/Ag_2.3%_ on CO_2_RR, a series of Cu_2_O/Ag NCs with different amounts of modified Ag were assessed for CO_2_RR performance (Supplementary Figs. 19 and 20). Characterizations underscore that all pristine Cu_2_O/Ag NCs with different compositions exhibit similar structures, whereas the density of deposited NPs and oxidation state on the surface of Cu_2_O NCs increased with the degree of modification (Supplementary Fig. 21). From the comparison of the potential for CO_2_RR of different catalysts at a high current density of 800 mA cm^−2^ (Fig. 4a), it can be seen that the demand potential decreases with increased Ag. This finding confirms the positive impact of Ag modification on CO_2_RR. The degree of introduced Ag-dependent FEs of products from these catalysts was assessed under the same current density. As is shown in Fig. 4b, c, increasing Ag in dCu_2_O/Ag leads to a volcano-shape for FE_EtOH_ that corresponds with a reverse-volcano on FE_CO_, and contrasts with the monotonously decreased FE for C_2_H_4_. This correlation between the amount of modified Ag and FEs for CO and EtOH was observed also with other applied currents (Supplementary Fig. 22). Common to Cu-based catalysts, the coverage of CO on Cu surface is conducive to C–C coupling to impact EtOH and C_2_H_4_ generation concurrently^24^. However, here for the dCu_2_O/Ag, only the FE for EtOH exhibits the dependent correlation with CO. These results indicate that the boosted EtOH is not only dependent on variable CO coverage on dCu_2_O/Ag, but also related to other unknown factors.

The stability of dCu_2_O/Ag_2.3%_ was evaluated via long-term chronopotentiometry testing. It was found that there is no apparent decay of activity with 6 h continuous operation, in which the selectivity of EtOH decreased ~6% following CO_2_RR (Supplementary Fig. 23). TEM image and XRD pattern of spent dCu_2_O/Ag_2.3%_ showed that morphology and structure are maintained following the stability test (Supplementary Fig. 24). Given the superior EtOH production on dCu_2_O/Ag_2.3%_, it was consequently assessed in a commercially relevant membrane electrode assembly (MEA) (Supplementary Fig. 25). It was found that there is a good, linear relationship between applied current and voltage in the MEA (Fig. 4e). The FEs for all products tested in flow cell electrolyzer were well-reproduced in MEA (Supplementary Fig. 26 and Fig. 4d), identifying that high current and high EtOH selectivity can be maintained under commercially relevant conditions (Fig. 4e). Importantly, the durability of CO_2_RR in the catholyte–free MEA significantly outperformed the flow cell electrolyzer, which exhibited a decrease of FE_EtOH_ (~3%) in 12 h operation under a full–cell voltage of −4.72 V with a total current density 800 mA cm^−2^, evidencing the good stability of dCu_2_O/Ag_2.3%_ for CO_2_RR (Fig. 4f).

### Mechanistic studies

The CO reduction reaction (CORR) on dCu_2_O/Ag was assessed to identify whether boosted C–C coupling and EtOH generation followed a classic CO–tandem mechanism (Supplementary Fig. 27). To permit a direct comparison, the CORR behavior of dCu_2_O and dCu_2_O/Au_2.3%_ were evaluated and the catalysts were activated by a similar process with CO_2_RR. It was expected that if the CO–tandem mechanism dominated, the CORR performance for the catalysts would be similar^28^. However, as is shown in Supplementary Fig. 28, the dCu_2_O/Ag_2.3%_ exhibits significant suppression of H_2_ and promoted C–C coupling for C_2+_ products under CORR. This is in significant contrast to dCu_2_O/Au_2.3%_ and dCu_2_O results. The partial current density of C_2+_ products for dCu_2_O/Ag_2.3%_ reaches 696.0 mA cm^−2^ at –1.56 V_RHE_ for CORR, and is significantly greater than those for dCu_2_O/Au_2.3%_ (~154.0 mA cm^−2^) and dCu_2_O (~188.0 mA cm^−2^). Similarly, the ratio FE_EtOH_/FE_C2H4_ for dCu_2_O/Ag_2.3%_ is also greater than that for dCu_2_O/Au_2.3%_ and dCu_2_O under CORR. Significantly, these findings from dCu_2_O/Ag_2.3%_ contrast with those for reported Cu–Ag catalyst with CO–tandem mechanism. This is interpreted that Ag modification results in intrinsic property changes in Cu active sites to: (1) suppress HER, (2) improve C–C coupling activity, and; (3) boost EtOH selectivity. In addition, the modification induced compressive strain, and morphology effects (crystal facets) for boosted EtOH production can be also excluded in our circumstances, because the exposed facets, surface structure and Cu–Cu bond length of both dCu_2_O/Au_2.3%_ and dCu_2_O/Ag_2.3%_ catalysts are similar. Therefore, other potential mechanisms on dCu_2_O/Ag_2.3%_ need to be assessed for boosted CO_2_RR performance.

Reaction pathways for C_2_H_4_ and EtOH production are similar on Cu surfaces, as they initiate with two adsorbed–CO dimerization followed by several steps of protonation and dehydration to generate a shared intermediate, ^*^HCCOH^3,14^. The selectivity between C_2_H_4_ and EtOH, is significantly dependent on the relative stabilities of the next-intermediates for EtOH and C_2_H_4_ pathways branched from ^*^HCCOH on Cu sites^3,14,40^. Cu with a relatively low coordinated surface and optimal oxide state is favorable for EtOH generation over C_2_H_4_ because the reaction intermediates for EtOH are more saturated compared with those for C_2_H_4_^41^, and the existing oxidation is feasible to binding of key oxygen-bound intermediates for EtOH generation^33,42^. Combined with this and previous experiment results, it is hypothesized that Ag-induced the moderate coordinated surface and optimal oxidation of Cu in dCu_2_O/Ag_2.3%_ are responsible for boosted EtOH selectivity. Therefore, the CO_2_RR intermediates chemisorbed on dCu_2_O, dCu_2_O/Ag_2.3%_, and dCu_2_O/Au_2.3%_ at different potentials were assessed via in situ attenuated total reflectance infrared absorption spectroscopy (ATR–IRAS) to determine the mechanism for boosted EtOH.

**Fig. 5:**
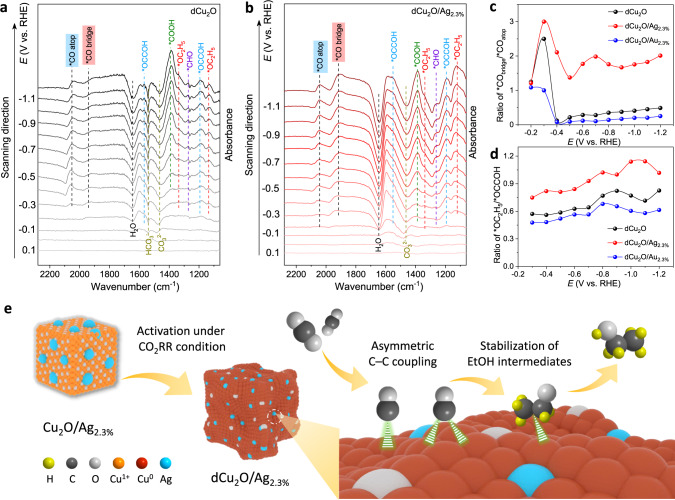
In situ ATR–IRAS measurement and mechanism for dCu_2_O/Ag_2.3%_. In situ ATR–IRAS obtained during chronopotentiometry in a potential window 0.2 to −1.2 V_RHE_ for **a** dCu_2_O, and **b** dCu_2_O/Ag_2.3%_ under CO_2_RR. (A reference spectrum obtained at 0.3 V_RHE_ in 1 M KOH is subtracted). Potential dependence of ratio of **c** ^*^CO_bridge_/^*^CO_atop_ and **d** ^*^OC_2_H_5_/^*^OCCOH obtained for dCu_2_O, dCu_2_O/Ag_2.3%_ and dCu_2_O/Au_2.3%_. **e** Schematic for boosted EtOH generation over dCu_2_O/Ag_2.3%_. Yellow-color, gray, white, orange, red, and azure spheres in the model represent H, C, O, Cu^1+^, Cu^0^, and Ag atoms, respectively.

As is shown in Fig. 5a, b and Supplementary Fig. 29, with the cathode potential at −0.3 V_RHE_, the ATR–IRAS spectra for these catalysts exhibit several new peaks. These are assigned to corresponding intermediates based on independently reported studies (Supplementary Table 4). In particular, there appear two peaks at 2044 and 1923 cm^−1^ for dCu_2_O/Ag_2.3%_, which are associated with the atop–adsorbed ^*^CO (^*^CO_atop_) and bridge–adsorbed ^*^CO (^*^CO_bridge_) on Cu surface, respectively^43–45^. In comparison, dCu_2_O and dCu_2_O/Au_2.3%_ mainly show the ^*^CO_atop_ peak with little evidence of ^*^CO_bridge_ binding in the same potential region. The different ^*^CO binding configurations on these catalysts can also be observed from CO temperature-programmed desorption (CO–TPD) (Supplementary Fig. 30b). Mathematical integration and statistical analyses confirm the ratio ^*^CO_bridge_/^*^CO_atop_ for dCu_2_O/Ag_2.3%_ is significantly greater than that for dCu_2_O or dCu_2_O/Au_2.3%_ over the potential range (Supplementary Fig. 31 and Fig. 5c). These findings evidence that compared with dCu_2_O and dCu_2_O/Au_2.3%_, the moderate coordination numbers and optimal oxidation for Cu surface in dCu_2_O/Ag_2.3%_ result in tailored ^*^CO configuration. Given the different electron back-donating and proton-combining ability of adsorbed ^*^CO on atop and bridge Cu sites, the energy barrier for following ^*^CO protonation is altered. Previous studies demonstrate that ^*^CO_bridge_ protonation is more energetically favorable than that of ^*^CO_atop_ on Cu surface^46^. Therefore, the C–C coupling on dCu_2_O/Ag_2.3%_ could be triggered under asymmetry between ^*^CO and ^*^CHO (or ^*^COH) following the ^*^CO_bridge_ protonation step. Notably, adsorbed ^*^CHO intermediate was observed on dCu_2_O/Ag_2.3%_ from ATR-FTIR spectra, and increased with the applied potential (Fig. 5b), strongly evidencing this process. This asymmetric C−C coupling has a lower energy barrier than that for *CO dimerization as evidenced by the reported theory studies^19^, which contributes to increased C_2+_ production.

Additional peaks from ATR–IRAS spectra at ~1567 and ~1182 cm^−1^ and ~1336 and ~1117 cm^−1^ were analyzed. These peaks, indexed to the absorbed ^*^OCCOH and ^*^OC_2_H_5_ on these catalysts, exhibit a ratio value for ^*^OC_2_H_5_/^*^OCCOH on dCu_2_O/Ag_2.3%_ that is significantly greater compared with that for dCu_2_O and dCu_2_O/Au_2.3%_. This finding evidences that the key ^*^OC_2_H_5_ intermediates for EtOH production are more stable on dCu_2_O/Ag_2.3%_ (Fig. 5d)^42,43^. This is attributed to the asymmetric C−C coupling induced unbalanced coordination environment, which disrupts the coordination sites for C_2_H_4_ intermediates, and thereby, stabilizes the EtOH intermediates. This is in agreement with report that diversity of ^*^CO binding site enhances C_2+_ liquid product formation^27^. Therefore we hypothesize that triggered asymmetric C−C coupling on dCu_2_O/Ag_2.3%_ boosts C_2+_ production and favors EtOH pathway via stabilizing pivotal intermediates.

In addition, the peak for the absorbed bicarbonate at 1547 cm^−1^ on dCu_2_O/Ag_2.3%_ is missing compared with dCu_2_O and dCu_2_O/Au_2.3%_, confirming that the value of the local pH on the electrode of dCu_2_O/Ag_2.3%_ is greater than that for dCu_2_O and dCu_2_O/Au_2.3%_ during CO_2_RR^47^. The high pH at the surface of electrode is thought to favor C–C coupling by lowering the energy barrier of CO_2_ activation and suppressing H_2_ evolution, and contributes to the boosted activity for CO_2_RR. Moreover, the CO–TPD and CO_2_–TPD for dCu_2_O/Ag_2.3%_ also exhibit a higher temperature for CO_2_ and CO desorption than those for dCu_2_O and dCu_2_O/Au_2.3_, indicating dCu_2_O/Ag_2.3%_ has stronger bonding strength of CO_2_ and CO for efficient CO_2_RR at large current (Supplementary Fig. 30).

Accordingly, based on the ATR–IRAS spectra analysis, the mechanism for boosted EtOH on dCu_2_O/Ag_2.3%_ can be soundly proposed (Fig. 5e). At first, the coordinated pure-Cu surface is replaced with neighboring Ag atoms with modification by Ag in Cu_2_O and reduces these to CuAg alloy under CO_2_RR. Then, the Ag-induced moderate coordination numbers and optimal oxidation of Cu surface regulate binding strength of ^*^CO, to configure mixed ^*^CO_bridge_ and ^*^CO_atop_ adsorption that triggers asymmetric C–C coupling after ^*^CO_bridge_ protonation. Because of the relatively low oxygen affinity and unsaturated nature of the C_2_H_4_ intermediates compared with EtOH, the asymmetric C–C coupling provides an unbalanced coordination environment that is beneficial for EtOH intermediate formation and stabilization in lower energy than that for C_2_H_4_, and thereby promotes the pathway for EtOH.

## Discussion

In summary, an assessment of a newly synthesized, silver-modified copper-oxide catalyst has confirmed that the CO_2_RR to EtOH pathway is accelerated via triggering the asymmetric C–C coupling. An optimized dCu_2_O/Ag_2.3%_ exhibits a FE of 40.8% and EE_HF_ of 22.3% for EtOH production in flow cell, together with boosted EtOH partial current density of 326.4 mA cm^−2^ at −0.89 V_RHE_ (with an 85 % *iR* correction). In situ ATR–IRAS spectroscopy confirmed that boosted EtOH selectivity results from moderate coordinated surface and optimal oxidation state of the Cu sites that gives mixed ^*^CO_bridge_ and ^*^CO_atop_ configurations for asymmetric C–C coupling to stabilize the EtOH intermediates. This demonstrated understanding of the mechanism for electroreduction of CO_2_–to– EtOH contrasts with reported, classic CO–tandem catalysis. It can be practically used to significantly boost EtOH production.

## Methods

### Preparation of Cu_2_O, Cu_2_O/Ag, and Cu_2_O/Au NCs

In a typical preparation for Cu_2_O/Ag_2.3%_ NCs, 0.5 mL NaOH solution (1 M) and 0.5 mL Cu(NO_3_)_2_ solution (0.1 M) were added to a 30 mL vial with 9 mL water under vigorous stirring for 5 min at RT to give a blue-color Cu(OH)_2_ suspension. 27 mg AA was added to the vial under vigorous stirring. The suspension changed from blue color to yellow, confirming the formation of Cu_2_O NCs. Following stirring for 30 min, 0.1 mL AgNO_3_ solution (0.01 M) was added to the vial, and stirring continued for 30 min. Cu_2_O/Ag_2.3%_ NCs were obtained and resulting products were collected by centrifugation and washed with EtOH. Preparation of Cu_2_O and Cu_2_O/Au NCs was similarly conducted, but without the addition of AgNO_3_ and with the replacement of AgNO_3_ with HAuCl_4_•4H_2_O. Cu_2_O/Ag and Cu_2_O/Au NCs with different compositions were prepared by adding selected volumes of AgNO_3_ and HAuCl_4_•4H_2_O solution (Supplementary Table 5).

### Characterizations

TEM and HAADF–STEM were conducted on a FEI Tecnai F20 transmission electron microscope with an acceleration voltage 200 kV. Samples were prepared by dropping EtOH dispersions of the samples onto carbon-coated, Cu TEM grids using a pipette, and dried under ambient RT conditions. SEM images were taken with a HITACHI S–3700 cold–field emission scanning electron microscope operated at 15 kV. XRD patterns were collected on X’Pert–Pro MPD diffractometer (Netherlands PANalytical) with a Cu Kα X-ray source (*λ* = 1.540598 Å). XPS was determined with an SSI S–Probe XPS Spectrometer. The carbon peak at 284.6 eV was used as a reference to correct for charging effects. XAS data were collected at the TPS–44 A beamline of the National Synchrotron Radiation Research Center (NSRRC, Hsinchu, Taiwan) using a Si (111) quick-scanning monochromator, and processed according to standard procedures using the Demeter program package (Version 0.9.24).

### CO_2_RR test in flow cell

Electroreduction of CO_2_ was tested in a microfluidics flow cell that consisted of two electrolyte chambers (20 × 5 × 3, mm) and one gas chamber (20 × 5 × 5, mm)^48^. An anion exchange membrane (Fumasep FAB–PK–130) was placed between two electrolyte chambers for products separation and ionic conduction. Catalyst-deposited GDE, micro Ag/AgCl electrode (4.0 M KCl), and Ni-foam (0.5 mm thickness), respectively, served as working electrode, reference electrode, and anode. To fabricate the working electrode, a certain amount of catalysts (3 mg) were dispersed in 1 mL EtOH with 20 µL 5 wt% Nafion solution and then sprayed onto a gas diffusion layer (CeTech, NIS1007) via airbrush. The loading amount of catalysts on GDE was controlled to ~0.44 mg cm^−2^. The working electrode was placed between gas and catholyte chambers to ensure gaseous CO_2_ diffusion and reaction at the catholyte/catalysts interface. The reference electrodes were inserted in catholyte chamber and maintained at a specified distance with the working electrode. An electrochemical workstation (CHI660, Chenhua, Shanghai) with a current amplifier was used to perform the CO_2_RR test. 1 M KOH (20 mL) was circulated through the electrolyte chambers under constant flow (15 mL min^−1^) via peristaltic pump. CO_2_ was supplied into gas chambers by a mass-flow controller at a constant flow rate of 30 mL min^−1^. Reactions were tested via chronopotentiometry at differing currents for 1 h without *iR* correction. Gas and liquid products were analyzed, respectively, via GC (Agilent 8890) and ^1^H NMR (Agilent 600 MHz DirectDrive2 spectrometers).

Potentials were referenced to RHE and *iR* correction performed based on the following, namely:

1 $${{{{{{\rm{E}}}}}}}_{{{{{{\rm{RHE}}}}}}}={{{{{{\rm{E}}}}}}}_{{{{{{\rm{vs\; Ag}}}}}}/{{{{{\rm{AgCl}}}}}}}+0.059\,\times {{{{{\rm{pH}}}}}}+\,0.210+0.85\,\times \,{iR}$$

where *i* is the current at each applied potential and *R* the equivalent series resistance measured via electrochemical impedance spectroscopy in the frequency.

FE for the formation of CO_2_RR product was computed from:

2 $${{{{{\rm{FE}}}}}}={{{{{\rm{eF}}}}}}\times {{{{{\rm{n}}}}}}/{{{{{\rm{Q}}}}}}={{{{{\rm{eF}}}}}}\times {{{{{\rm{n}}}}}}/({{{{{\rm{I}}}}}}\times {{{{{\rm{t}}}}}})$$

in which e is the number of transferred electrons for each product, F the Faraday constant, Q charge, I applied current, t reaction time, and n total product (in mole).

EE_HC_ was computed on the basis of the cathodic CO_2_RR coupled with the anodic oxygen evolution reaction (O_2_ + 4H^+^ + 4e^−^ ↔ 2H_2_O; 1.23 V *vs* RHE) from:

3 $${{{{{{\rm{EE}}}}}}}_{{{{{{\rm{HC}}}}}}}=\frac{{E}_{{{{{{\rm{oe}}}}}}}^\circ -{E}_{{{{{{\rm{red}}}}}}}^\circ }{{E}_{{{{{{\rm{oe}}}}}}}-{E}_{{{{{{\rm{red}}}}}}}}\times {{{{{{\rm{FE}}}}}}}_{{{{{{\rm{EtOH}}}}}}}$$

where *E*_oe_^o^ and *E*_red_^o^ are, respectively, the thermodynamic potential for oxygen evolution and CO_2_RR to EtOH (0.08 V vs RHE), *E*_oe_ and *E*_red_ applied potentials at, respectively, anode and cathode. For the computation of the half-cell EE, the anodic reaction was assumed to occur with an overpotential of 0 V, that is, *E*_oe_ = 1.23 V.

### CO_2_RR test in MEA

Electroreduction of CO_2_ in MEA consisted of two titanium backplates (TA2 grade) with a 4.0 cm^2^ serpentine flow field, and MEA. Catalyst-deposited GDE (~0.44 mg cm^−2^ for Cu_2_O/Ag_2.3%_ NCs) and Ni-foam (0.5 mm thickness) were used, respectively, as cathode and anode. The cathode and anode were pressed onto sides of the anion exchange membrane (Sustainion 37–50, Dioxide Materials). The gap between the electrodes was minimized to reduce ohmic loss. Gaseous CO_2_ (30 mL min^−1^) was passed behind the GDL to contact the catalyst, and 0.1 M solution was used as the anolyte which was circulated via pump at 20 mL min^−1^. CO_2_RR performance for MEA was evaluated by applying different currents with a current amplifier in the two-electrode system at the CHI660 (Chenhua, Shanghai) electrochemical workstation. Cathodic gas products were vented through a simplified cold–trap to collect permeable liquid prior to gas chromatograph testing. FE values for the liquid products were computed based on the total mass of product collected on anode and cathode.

### ECSA measurement

ECSA was measured by double-layer capacitance (DLC) and Pb underpotential deposition (Pb UPD) methods. All experiments are conducted in the flow cell and the used catalysts are obtained following activation under CO_2_RR. For DLC method, Cyclic Voltammetry (CV) scans were conducted at the potential range from 0.15 to 0.20 V vs RHE with increasing scan rates of 10, 20, 40, 60, 80, and 100 mV s^−1^. The capacitance currents at 0.17 V vs RHE were plotted against the scan rates, and the double-layer capacitance (C_dl_, mF cm^−2^) was derived from the slope according to the following:

4 $${{{{{{\rm{C}}}}}}}_{{{{{{\rm{dl}}}}}}}={{{{{\rm{I}}}}}}/{{{{{\rm{v}}}}}}$$

where I is the capacitance current (half of the difference between the anodic current density and cathodic current density, (J_a_−J_c_)/2), and v is the scan rate.

For Pb UPD method, the CV scans were conducted in Ar-saturated 0.01 M HClO_4_ and 1 mM PbCl_2_ at the potential range from 0.15 to 0.20 V *vs* RHE with scan rates of 10 mV s^−1^. The ECSA of catalysts was determined according to the following:

5 $${{{{{{\rm{ECSA}}}}}}}_{{{{{{\rm{Pb}}}}}}\; {{{{{\rm{UPD}}}}}}}={{{{{{\rm{A}}}}}}}_{{{{{{\rm{Pb}}}}}}\; {{{{{\rm{UPD}}}}}}}/(320{{\upmu }}{{{{{\rm{C}}}}}}{{{{{{\rm{cm}}}}}}}^{-2}{{{{{\rm{v}}}}}})$$

where A_Pb UPD_ is the peak area of monolayer Pb stripping, v the scan rate and the constant 320 μC cm^−2^ is the charge density factor for the UPD of Pb on copper^33^.

### In situ ATR–IRAS measurement

In situ ATR–IRAS was performed on a Nicolet iS20 spectrometer equipped with an HgCdTe (MCT) detector and a VeeMax III (PIKE Technologies) accessory. The measurement was conducted in a homemade electrochemical single-cell furnished with Pt–wire and Ag/AgCl as counter and reference electrodes. A fixed-angle Ge prism (60°) coated with catalysts embed into the bottom of the cell served as the working electrode. Before testing, the detector was cooled with liquid nitrogen for at least 30 min to maintain a stable signal. Chronoamperometry was used for CO_2_RR test and was accompanied by the spectrum collection (32 scans, 4 cm^−1^ resolution). All spectra were subtracted with the background.

## Supplementary information

Supplementary Information

## Data Availability

Data that support findings from this study are available from the corresponding author on reasonable request.
