# Supplementary material for: Boosting electrocatalytic CO2–to–ethanol production via asymmetric C–C coupling
Source: Nat Commun. 2022 Jun 29;13:3754. doi: 10.1038/s41467-022-31427-9 (PMC9243136; doi:10.1038/s41467-022-31427-9)
Supplement: Supplementary file 1 — Supplementary Information [file 41467_2022_31427_MOESM1_ESM.pdf]

Supporting information for

Boosting electrocatalytic CO_2–to–ethanol production *via* asymmetric C–C coupling

Pengtang Wang[1,2#], Hao Yang[3#], Cheng Tang[1], Yu Wu[3], Yao Zheng[1], Tao Cheng[3], Kenneth Davey[1], Xiaoqing Huang[2*] and Shi-Zhang Qiao[1*]

[1]School of Chemical Engineering and Advanced Materials, The University of Adelaide, Adelaide, SA 5005, Australia

[2]State Key Laboratory of Physical Chemistry of Solid Surfaces, College of Chemistry and Chemical Engineering, Xiamen University, Xiamen, 361005, China

[3]Institute of Functional Nano & Soft Materials (FUNSOM), Jiangsu Key Laboratory for Carbon-Based Functional Materials & Devices, Joint International Research Laboratory of Carbon-Based Functional Materials and Devices, Soochow University, Suzhou, 215123, China

[#]These authors contributed equally to this work.

[*]Corresponding author e-mail: hxq006@xmu.edu.cn; s.qiao@adelaide.edu.au

Supplementary Results

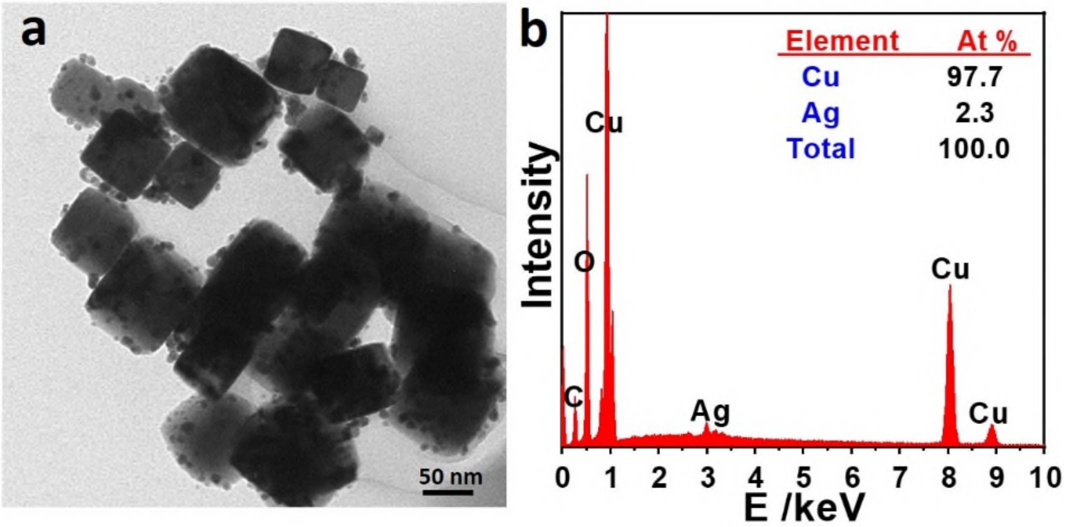

Supplementary Fig. 1 | Morphology and composition characterization of $Cu_2O/Ag_{2.3\%}$ NCs. (a) TEM image and (b) SEM-EDS pattern for $Cu_2O/Ag_{2.3\%}$ NCs.

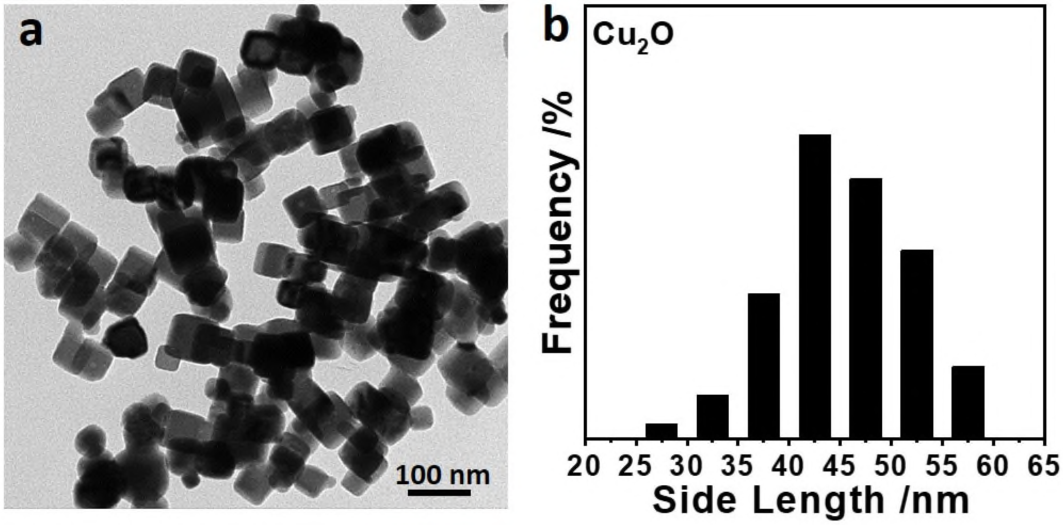

Supplementary Fig. 2 | Size distribution of Cu_2O NCs. (a) TEM image and (b) side length histogram for Cu_2O NCs.

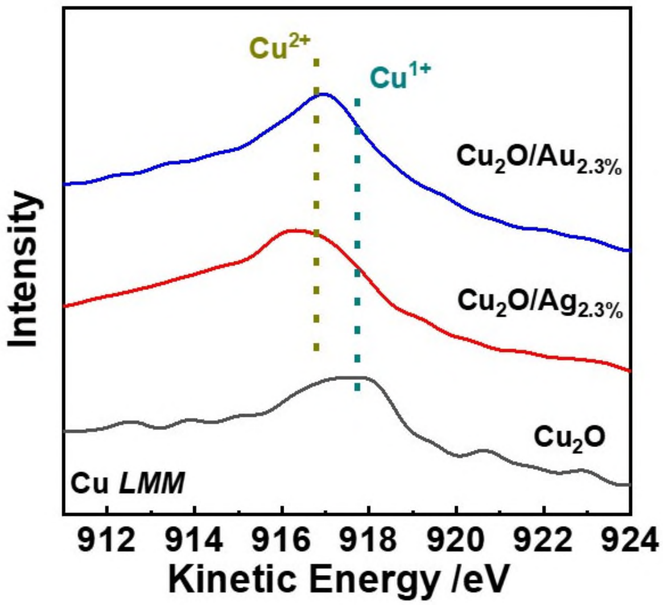

Supplementary Fig. 3 | Cu *LMM* AES curves for Cu_2O, $Cu_2O/Ag_{2.3\%}$ and $Cu_2O/Au_{2.3\%}$ NCs.

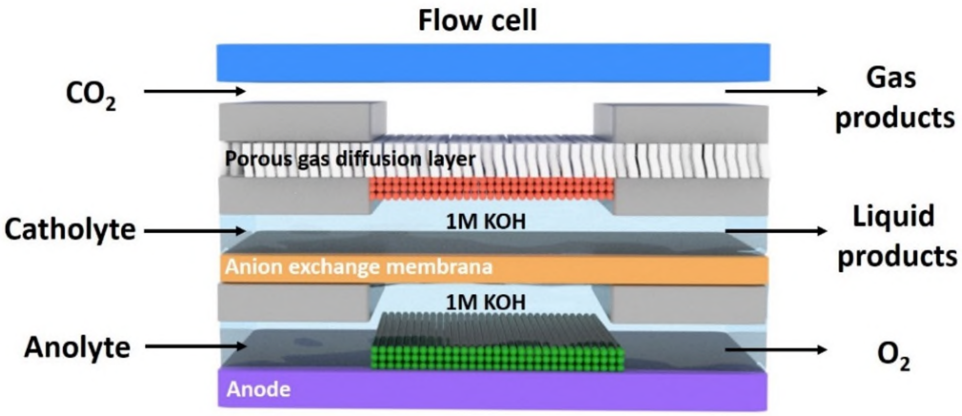

Supplementary Fig. 4 | Schematic for cathode flow cell for CO_2RR.

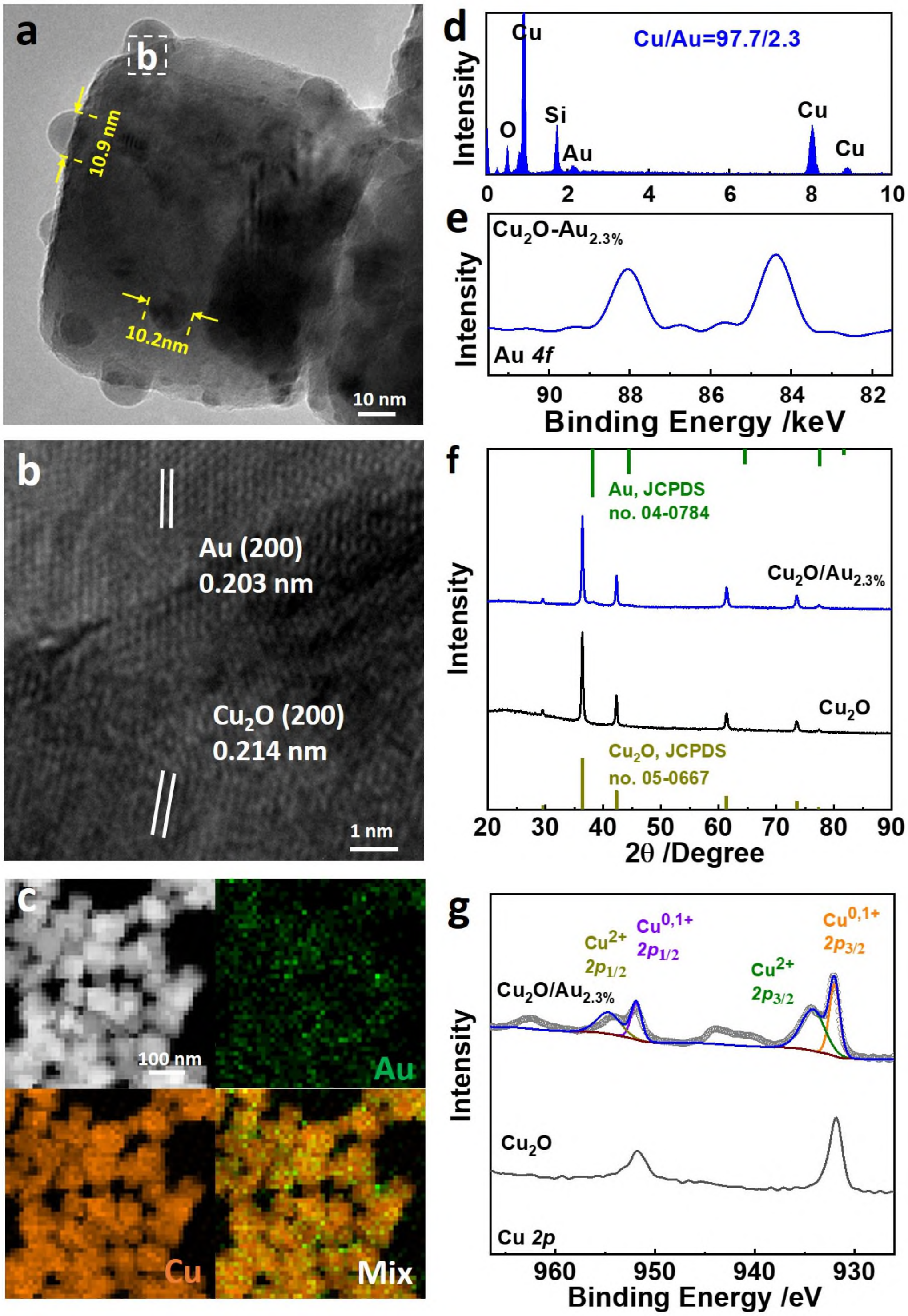

Supplementary Fig. 5 | Structural characterization of $Cu_2O/Au_{2.3\%}$ NCs. (a) TEM image, (b) HRTEM image, (c) EDS elemental mapping images, (d) SEM-EDS pattern and (e) Au *4f* XPS curve for $Cu_2O/Au_{2.3\%}$ NCs. (f) XRD pattern and (g) Cu *2p* XPS curves for Cu_2O and $Cu_2O/Au_{2.3\%}$ NCs.

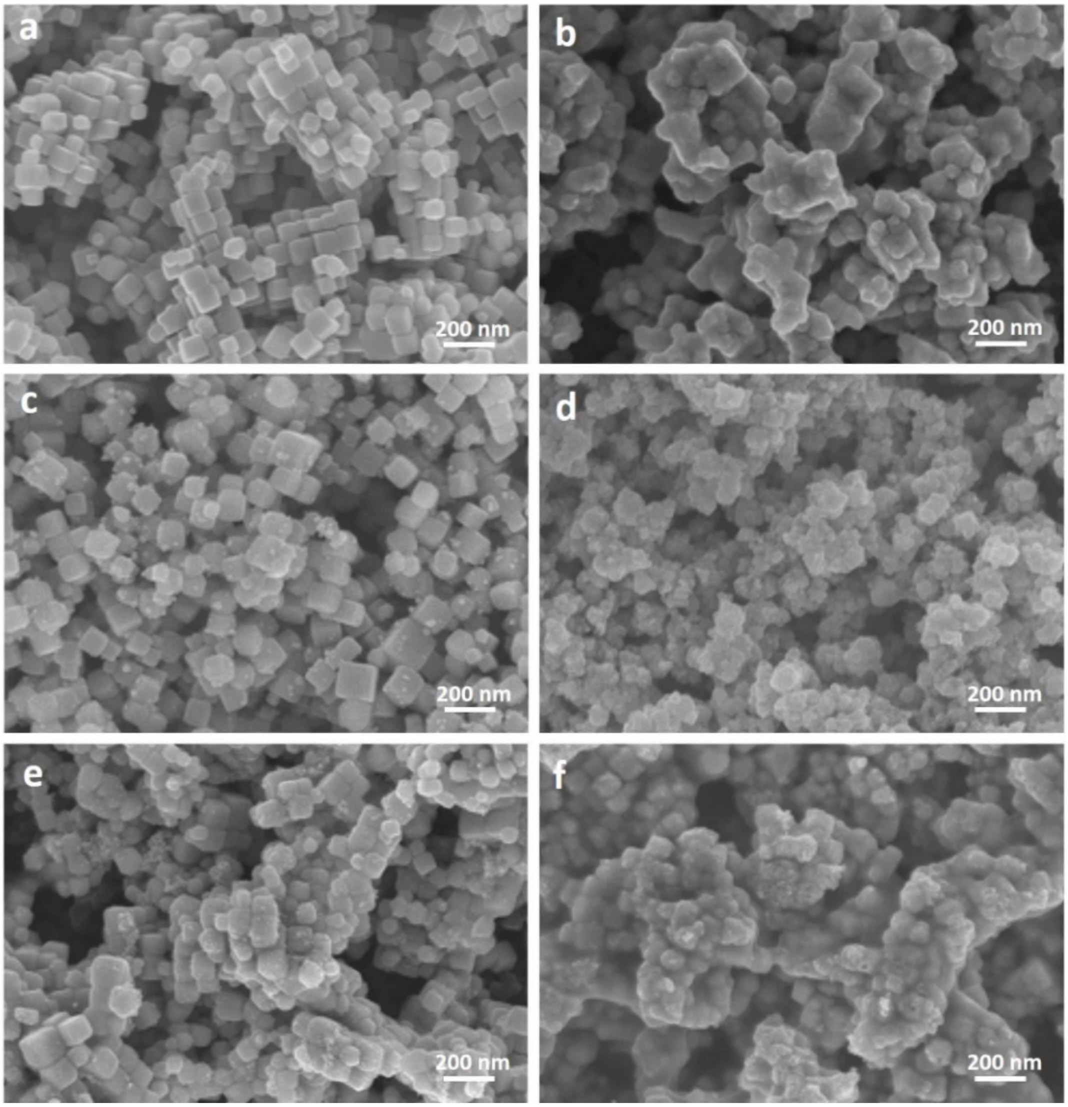

Supplementary Fig. 6 | SEM images of (a, b) Cu_2O, (c, d) $Cu_2O/Ag_{2.3\%}$ and (e, f) $Cu_2O/Au_{2.3\%}$ NCs (a, c, e) before and (b, d, f) following activation in CO_2 at 200 mA cm^{-2} for 30 min.

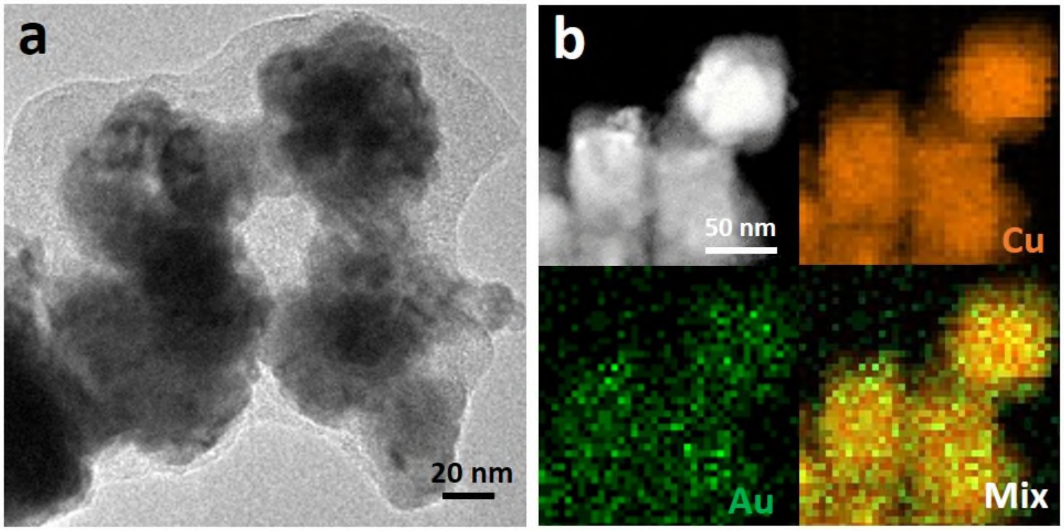

Supplementary Fig. 7 | (a) TEM image and (b) HAADF-STEM image with EDS elemental mapping of $dCu_2O/Au_{2.3\%}$.

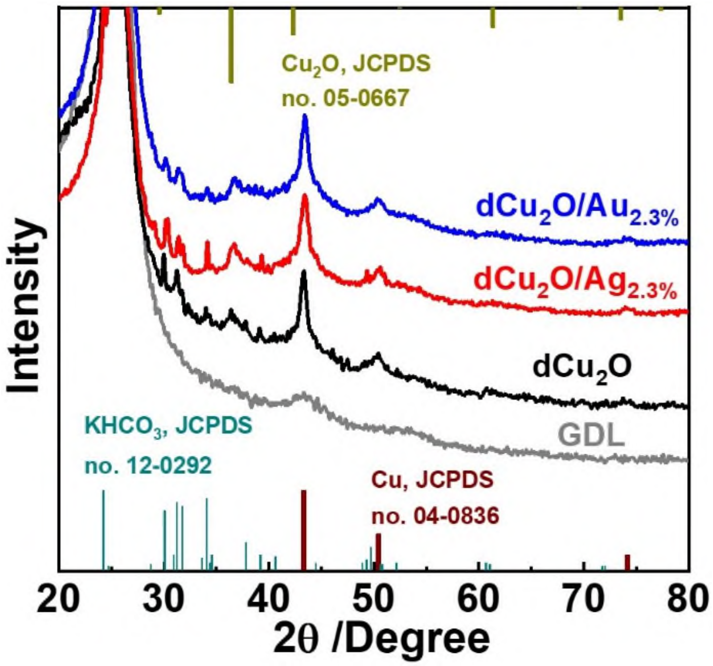

Supplementary Fig. 8 | XRD patterns for dCu_2O, $dCu_2O/Ag_{2.3\%}$ and $dCu_2O/Au_{2.3\%}$. All samples were measured on GDL.

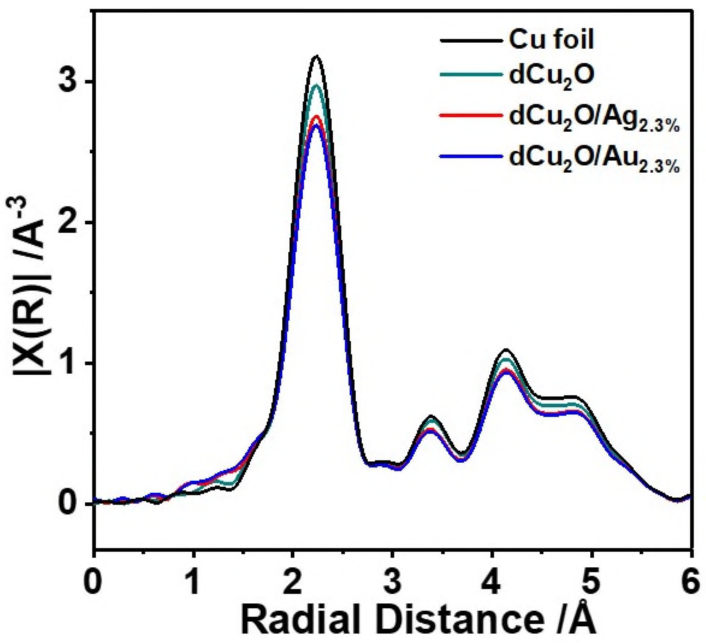

Supplementary Fig. 9 | *In situ* Cu K edge EXAFS of Cu-foil, dCu_2O, $dCu_2O/Ag_{2.3\%}$ and $dCu_2O/Au_{2.3\%}$.

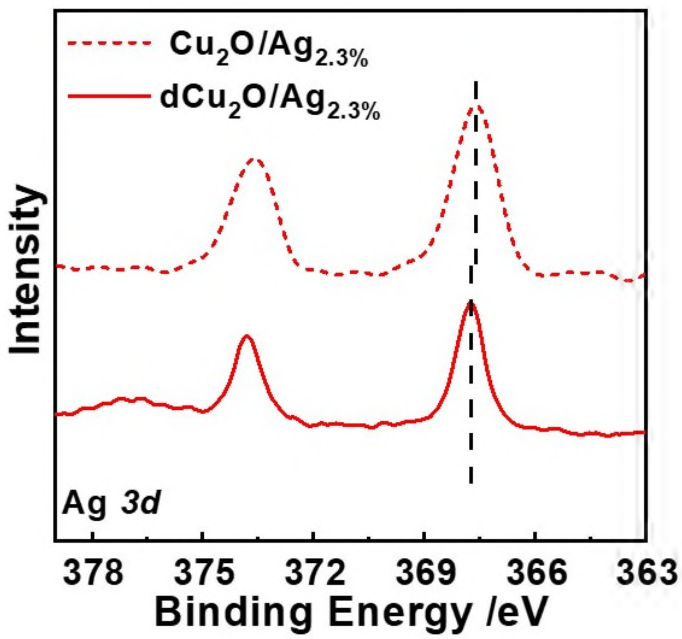

Supplementary Fig. 10 | Ag *3d* XPS curves of $Cu_2O/Ag_{2.3\%}$ and $dCu_2O/Ag_{2.3\%}$.

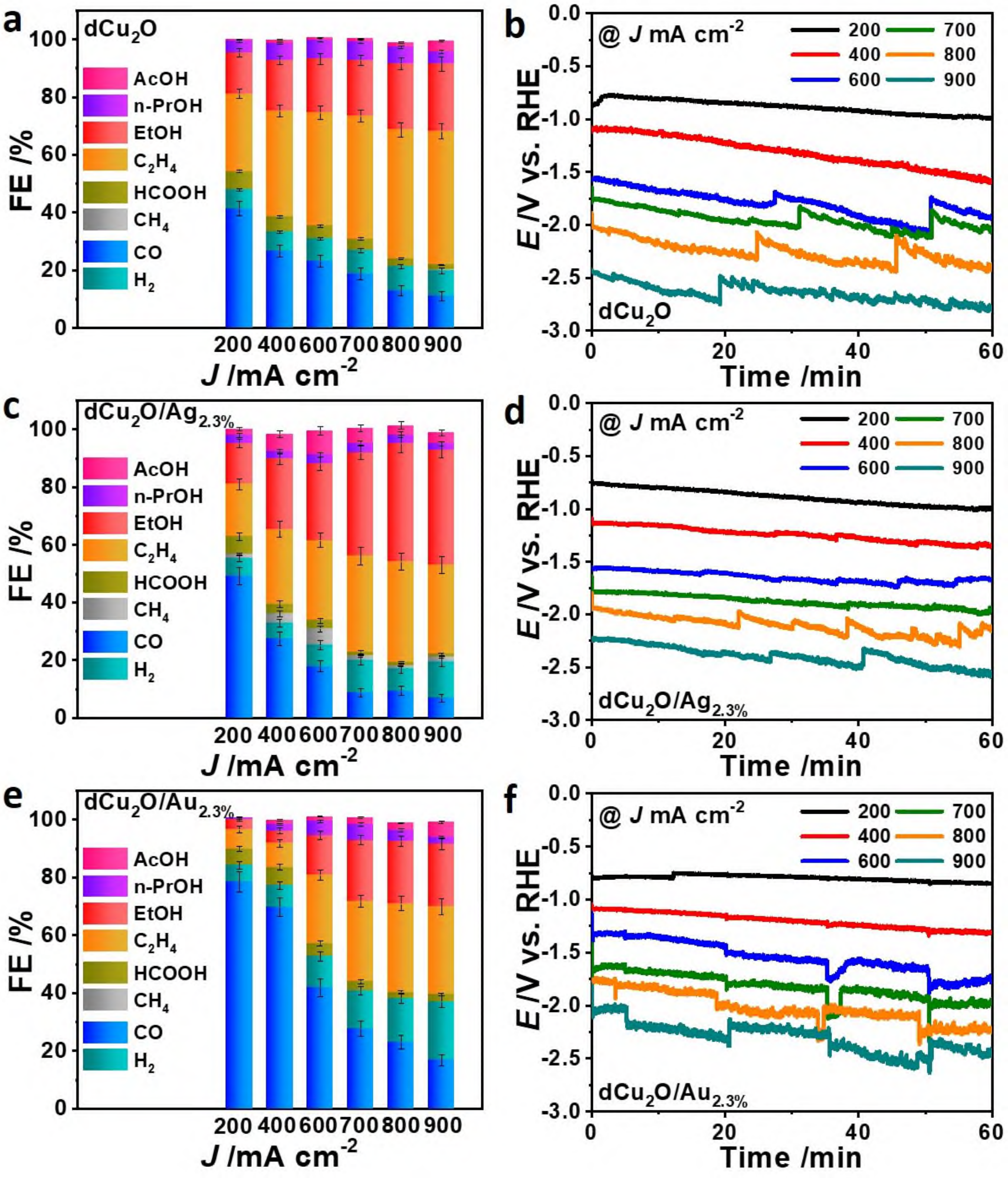

Supplementary Fig. 11 | (a, c, e) Product FEs and (b, d, f) Chronoamperometry curves for (a, b) dCu_2O, (c, d) $dCu_2O/Ag_{2.3\%}$ and (e, f) $dCu_2O/Au_{2.3\%}$ during CO_2RR at applied current. Error bars correspond to the standard deviation of three independent measurements.

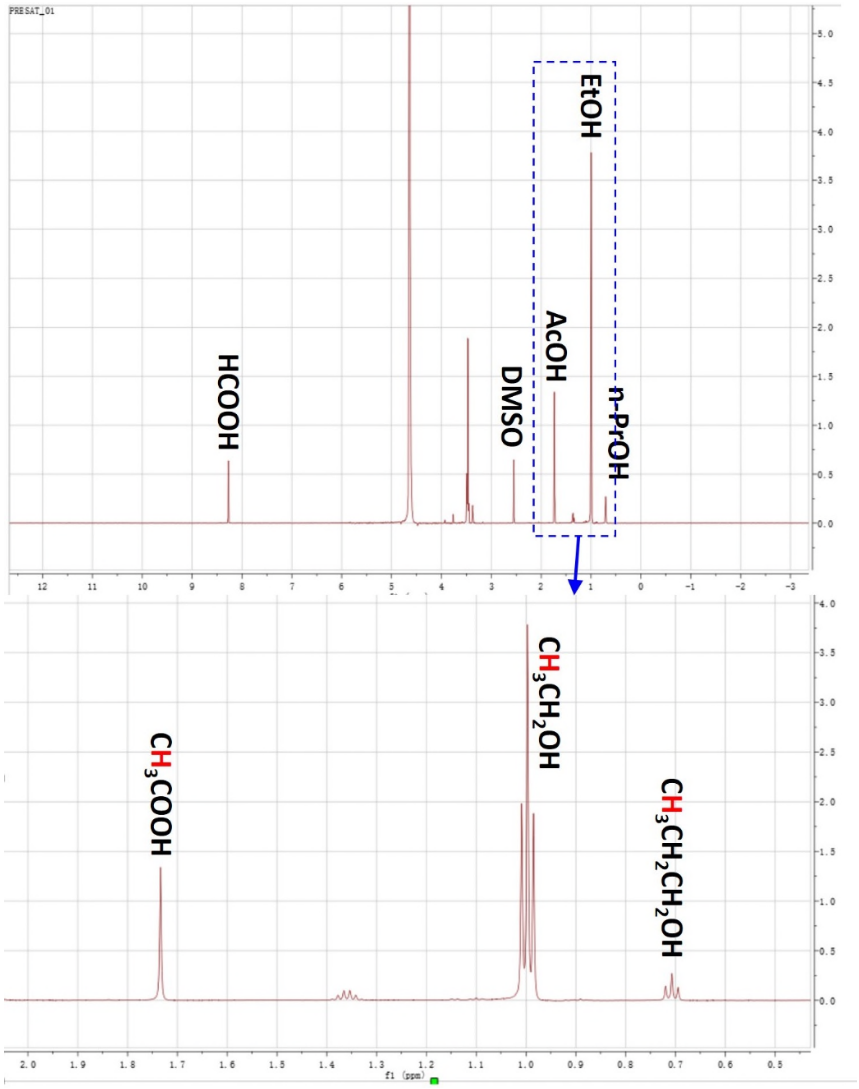

Supplementary Fig. 12 | Representative ^{1}H-NMR spectrum for catholyte following CO_2RR on dCu_2O/$Ag_{2.3\%}$ cathode applying current density of 800 mA cm^{-2} in 1 M KOH. DMSO is used as internal standard.

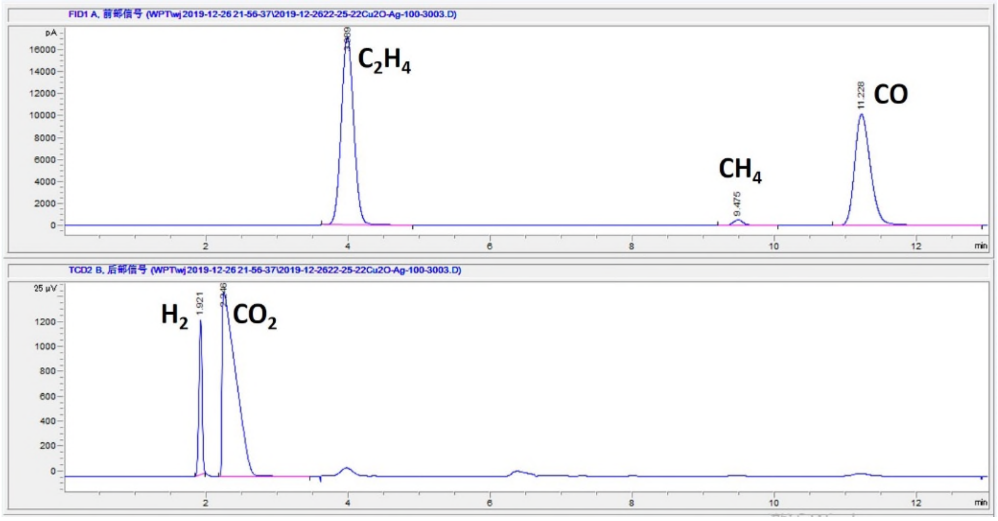

Supplementary Fig. 13 | GC trace of gaseous product during electrochemical CO_2RR at 800 mA cm^{-2} in 1 M KOH on $dCu_2O/Ag_{2.3\%}$.

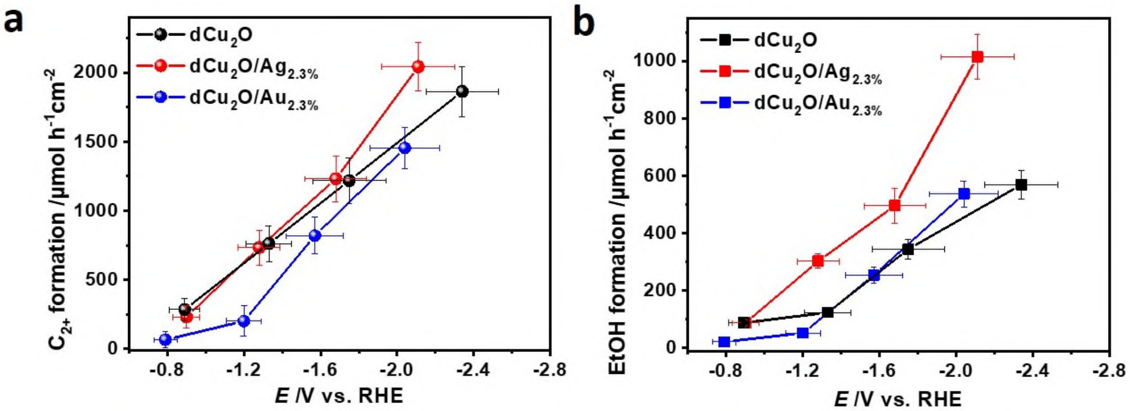

Supplementary Fig. 14 | (a) C_{2+} and (b) EtOH formation vs. potential referred to reversible hydrogen electrode (RHE) on dCu_2O, $dCu_2O/Ag_{2.3\%}$ and $dCu_2O/Au_{2.3\%}$. Error bars correspond to the standard deviation of three independent measurements.

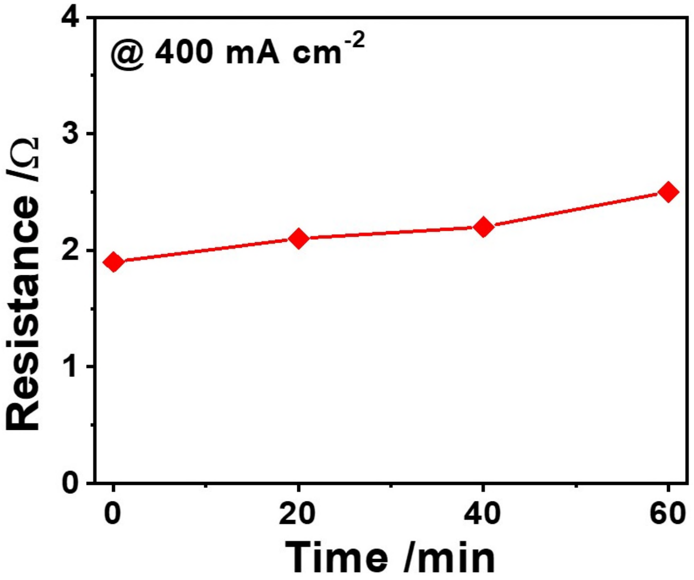

Supplementary Fig. 15 | Resistance between working and reference electrodes determined by EIS during electrolysis at 400 mA.

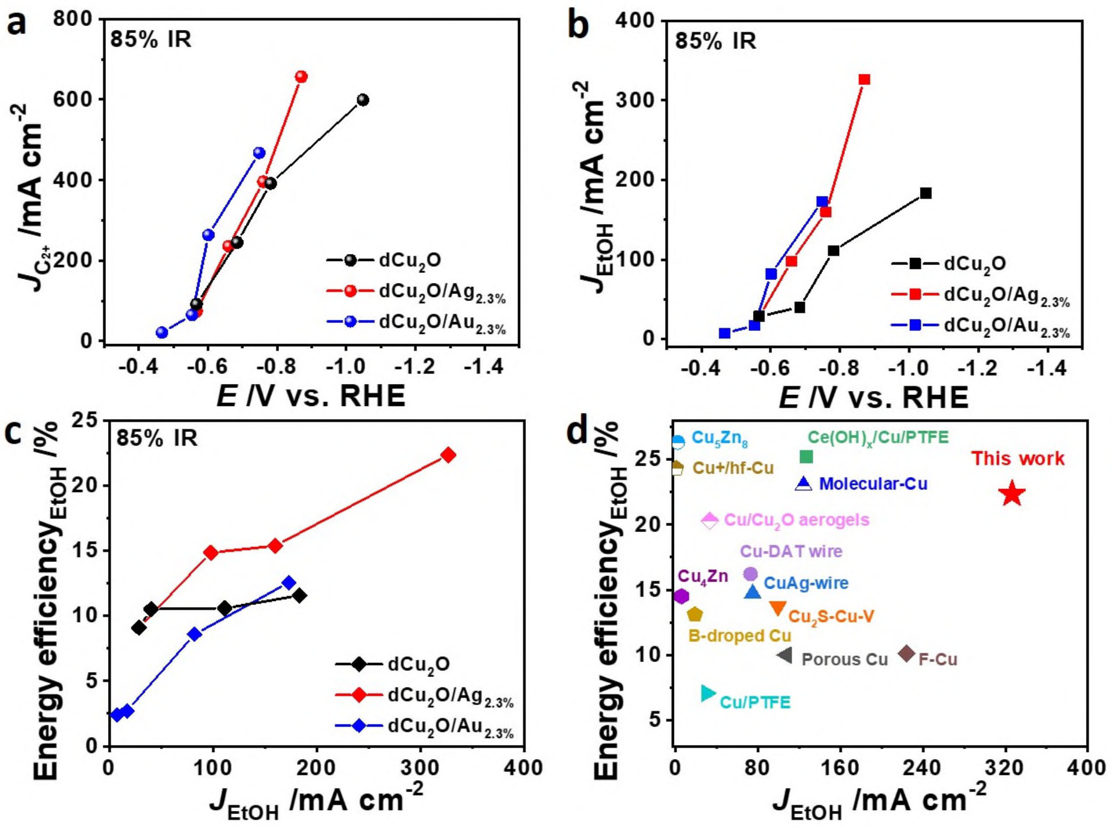

Supplementary Fig. 16 | Partial (a) C_{2+} and (b) EtOH current density vs. potential referred to reversible hydrogen electrode (RHE) with 85 % IR compensation on dCu_2O, $dCu_2O/Ag_{2.3\%}$ and $dCu_2O/Au_{2.3\%}$. (c) EtOH energy efficiency for CO_2RR on dCu_2O, $dCu_2O/Ag_{2.3\%}$ and $dCu_2O/Au_{2.3\%}$ under differing current density with 85 % IR compensation. (d) EtOH energy efficiency and current density of CO_2RR on selected Cu-based catalysts.

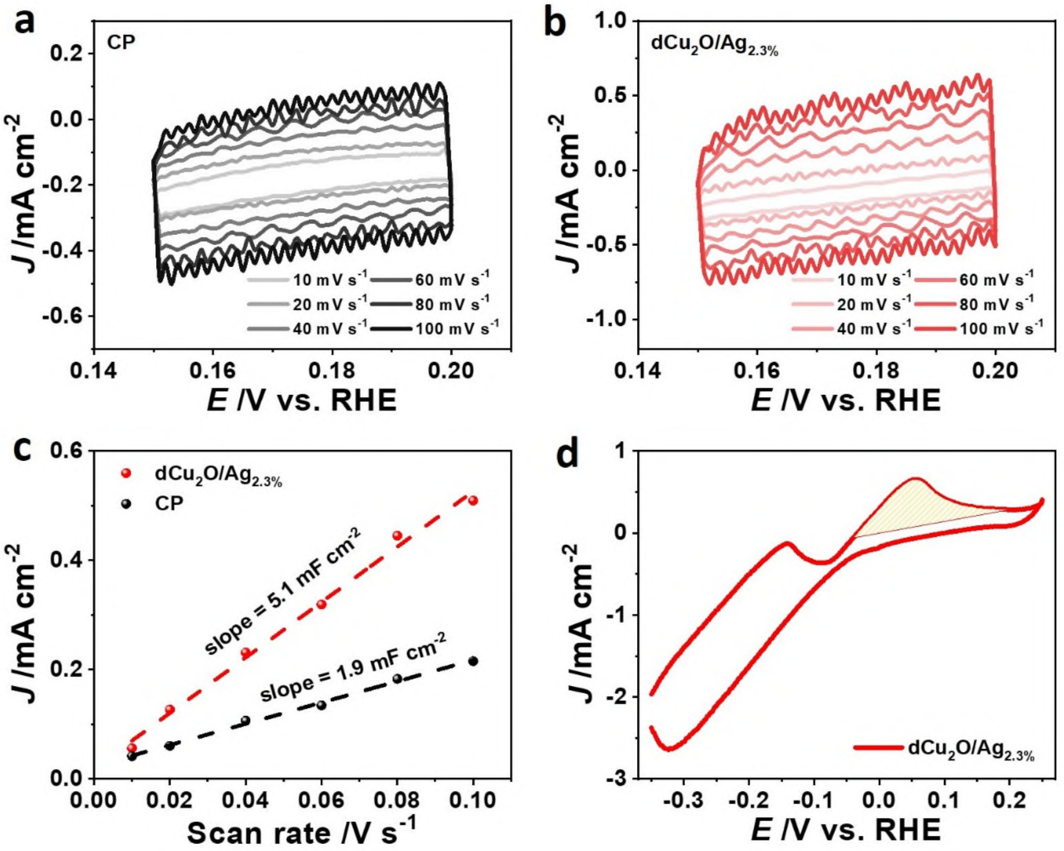

Supplementary Fig. 17. | ECSA measurement of $dCu_2O/Ag_{2.3\%}$. (a, b) CV curves for carbon paper and $dCu_2O/Ag_{2.3\%}$ obtained in capacitance region at varying scan rate. (c) Capacitance current density at 0.17 V vs. RHE as a function of scan rate. (d) CV curves for Pb stripping for $dCu_2O/Ag_{2.3\%}$ obtained in Ar saturated 0.01 M $HClO_4$ + 1 mM $PbCl_2$ solution at a scan rate of 10 mV s^{-1}. The highlighted yellow-color peak represents monolayer Pb stripping on $dCu_2O/Ag_{2.3\%}$.

Note: Given that the gas diffusion layer (carbon paper) will contribute to capacitance to impact the real capacitance for $dCu_2O/Ag_{2.3\%}$ ($C_{dl(dCu2O/Ag2.3\%)}$), the real ECSA for $dCu_2O/Ag_{2.3\%}$ was therefore computed by subtracting $C_{dl(carbon\ paper)}$ from $C_{dl(dCu2O/Ag2.3\%)}$, and determined to be 3.2 mF cm^{-2}.

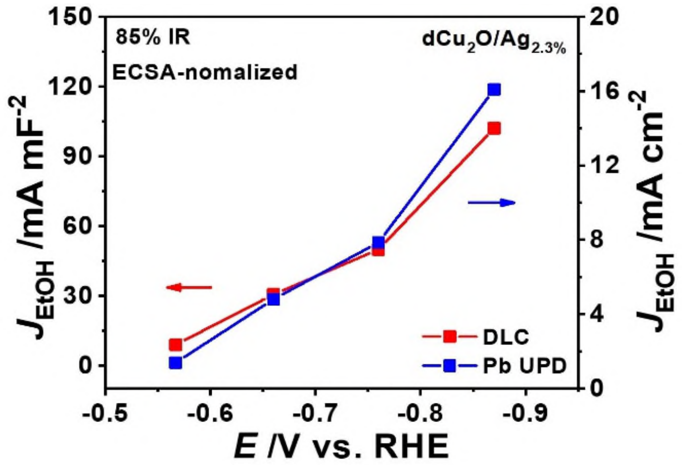

Supplementary Fig. 18 | ECSA-normalized EtOH current density under different potential of $dCu_2O/Ag_{2.3\%}$. The ECSAs were obtained with DLC and Pb UPD method, respectively.

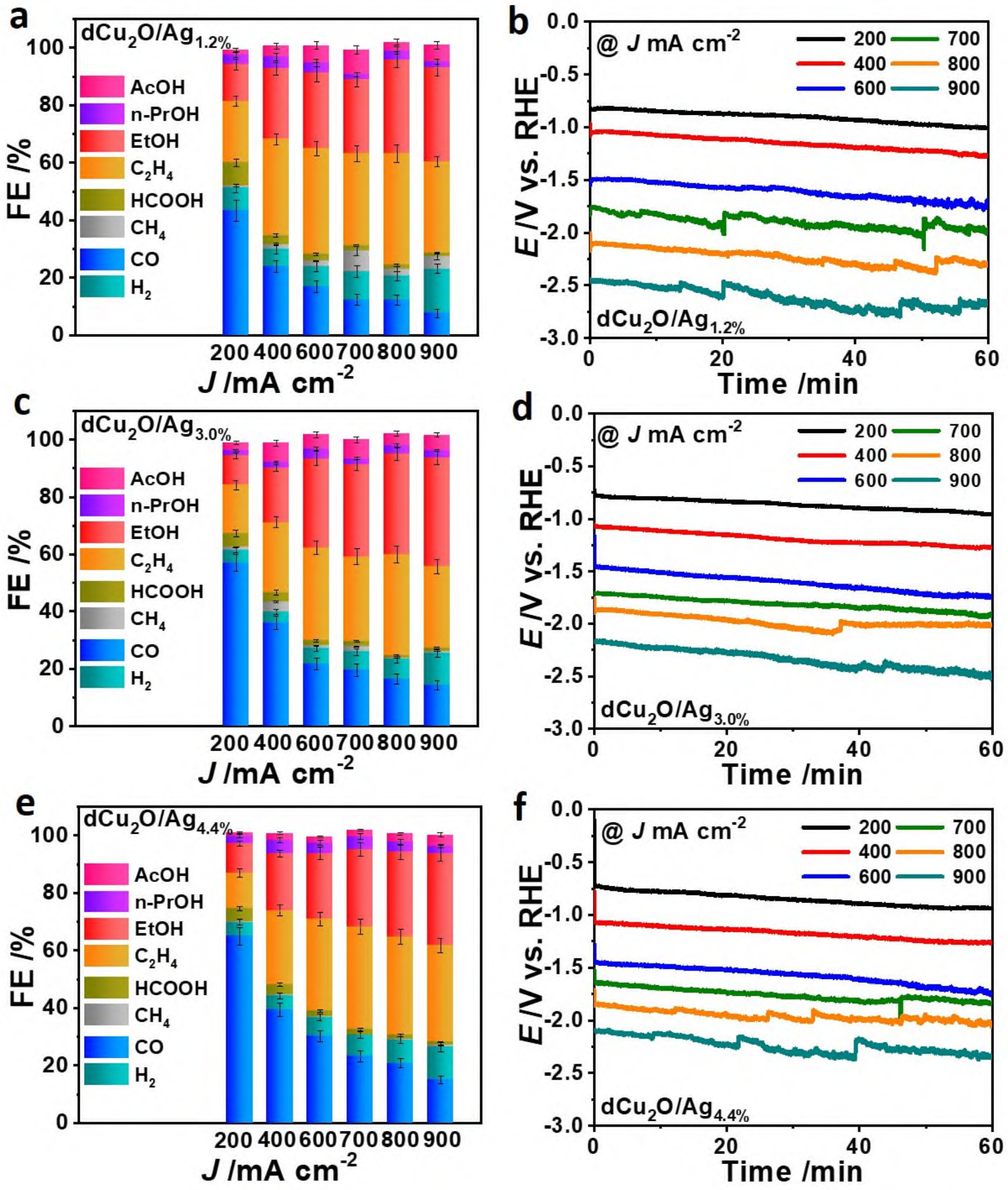

Supplementary Fig. 19 | (a, c, e) Product FEs and (b, d, f) Chronoamperometry curves for (a, b) $dCu_2O/Ag_{1.2\%}$, (c, d) $dCu_2O/Ag_{3.0\%}$ and (e, f) $dCu_2O/Ag_{4.4\%}$ during CO_2RR at applied current. Error bars correspond to the standard deviation of three independent measurements.

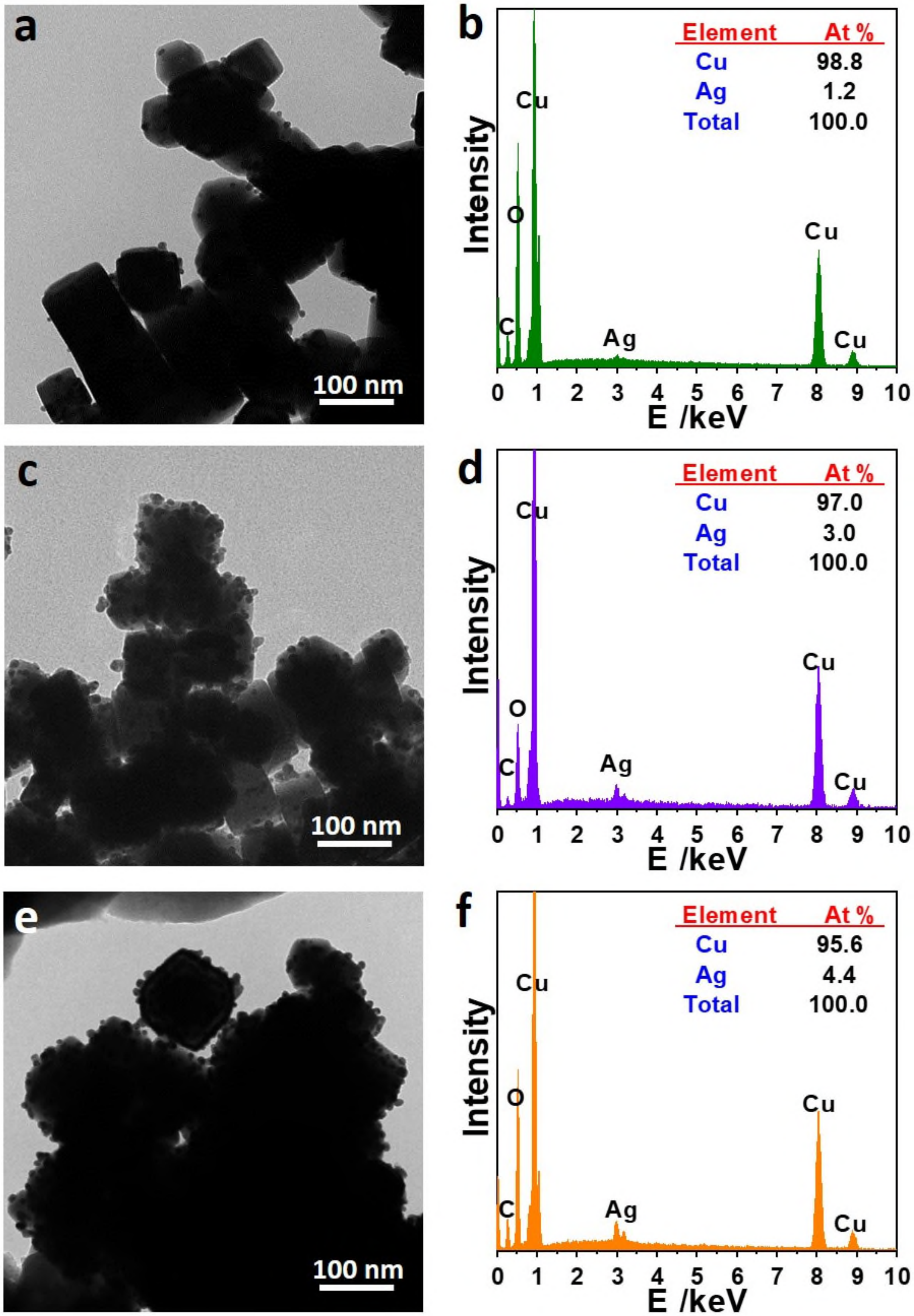

Supplementary Fig. 20 | Morphology and composition characterization of Cu_2O/Ag NCs with different Ag modification. (a, c, e) TEM images and (b, d, f) EDS patterns for (a, b) $Cu_2O/Ag_{1.2\%}$, (c, d) $Cu_2O/Ag_{3.0\%}$ and (e, f) $Cu_2O/Ag_{4.4\%}$ NCs.

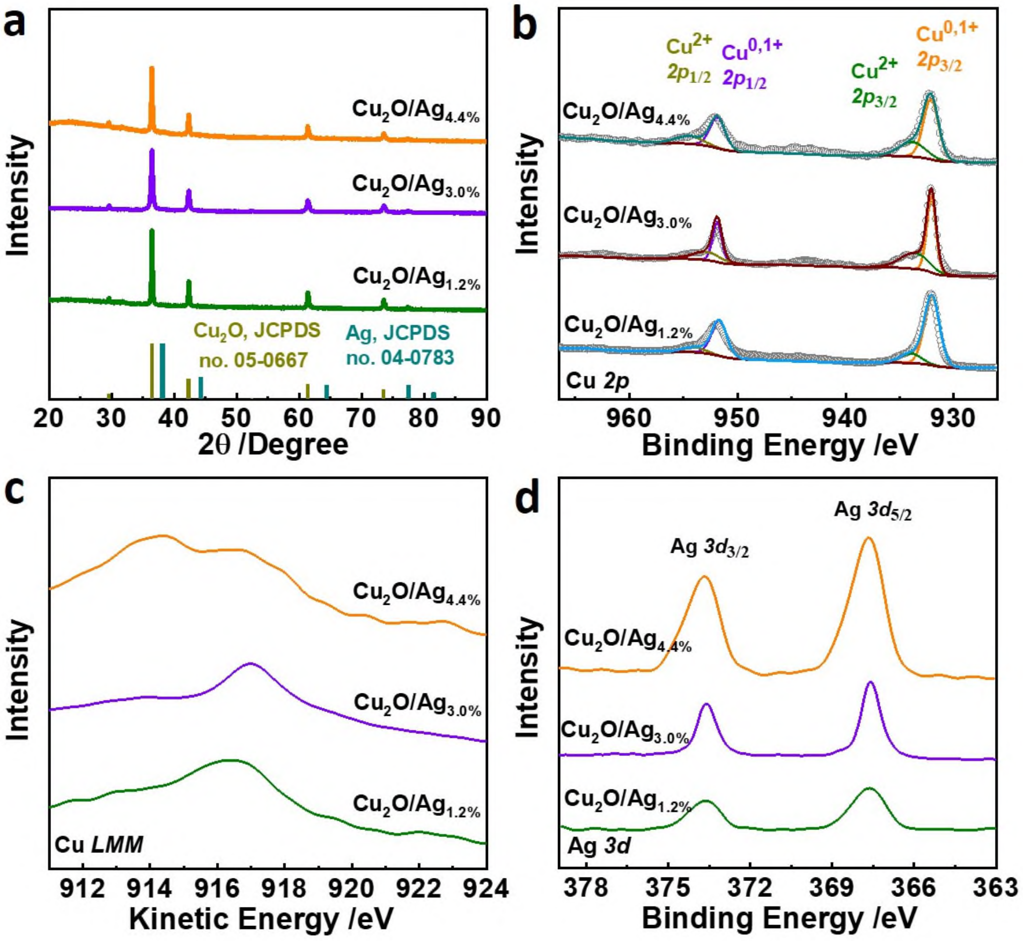

Supplementary Fig. 21 | Phase and valence state analysis of Cu_2O/Ag NCs with different Ag modification. (a) XRD patterns, (b) Cu *2p* XPS curves, (c) Cu *LMM* AES curves and (d) Ag *3d* XPS curves for $Cu_2O/Ag_{1.2\%}$, $Cu_2O/Ag_{3.0\%}$ and $Cu_2O/Ag_{4.4\%}$ NCs.

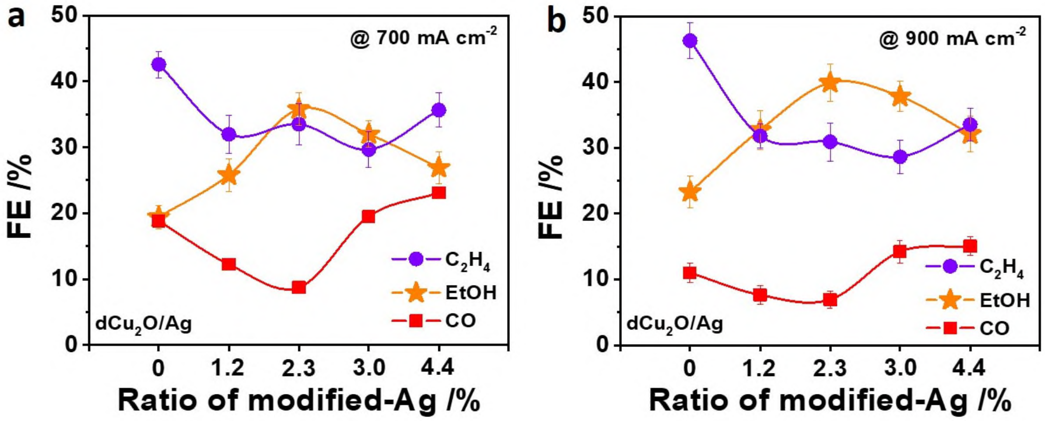

Supplementary Fig. 22 | Product FEs for CO, C_2H_4 and EtOH on Cu_2O/Ag NCs with modified Ag at current density (a) 700 and (b) 900 mA cm^{-2}.

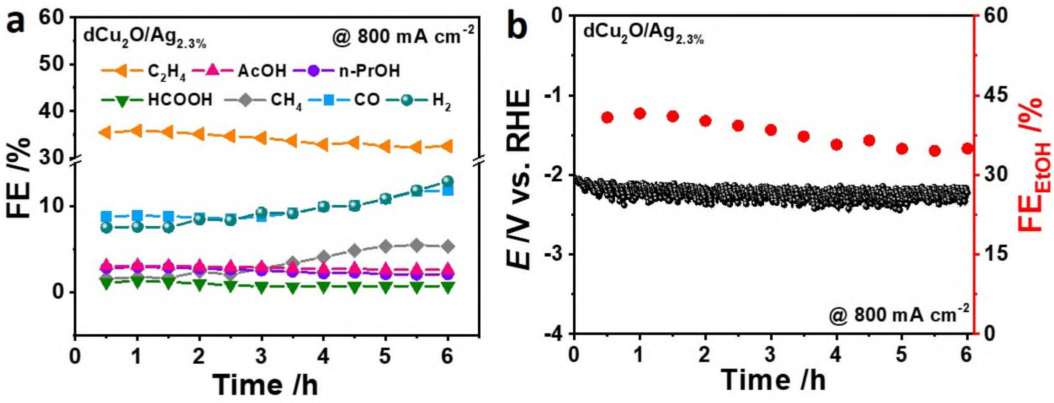

Supplementary Fig. 23 | CO_2RR stability test of $dCu_2O/Ag_{2.3\%}$ in flow cell. (a) FEs for H_2, CO, CH_4, C_2H_4, HCOOH, AcOH and PrOH for $dCu_2O/Ag_{2.3\%}$ at current density 800 mA cm^{-2} during CO_2RR stability test. (b) FE for C_2H_5OH and chronopotentiometry curve for $dCu_2O/Ag_{2.3\%}$ at current density 800 mA cm^{-2} in CO_2RR stability test.

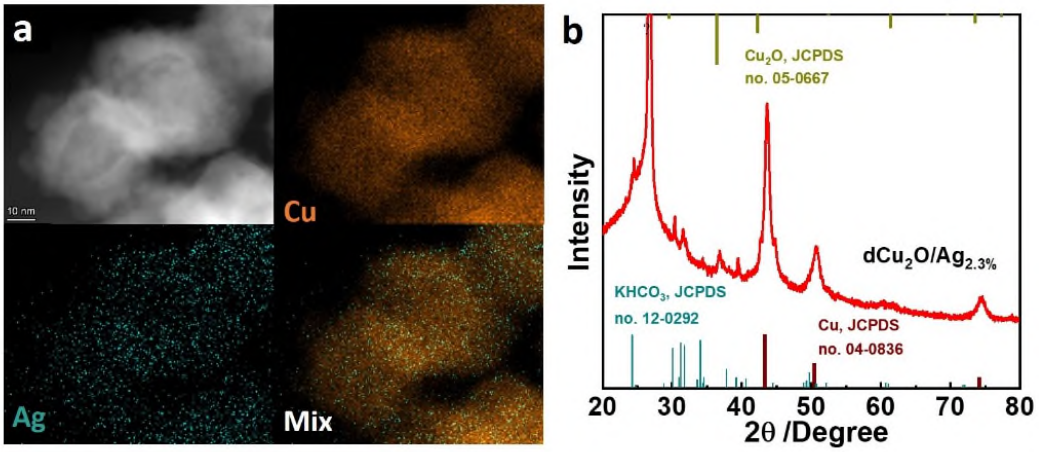

Supplementary Fig. 24 | (a) HAADF-STEM image with EDS elemental mappings and (b) XRD pattern for $dCu_2O/Ag_{2.3\%}$ following 6 h stability test in flow cell.

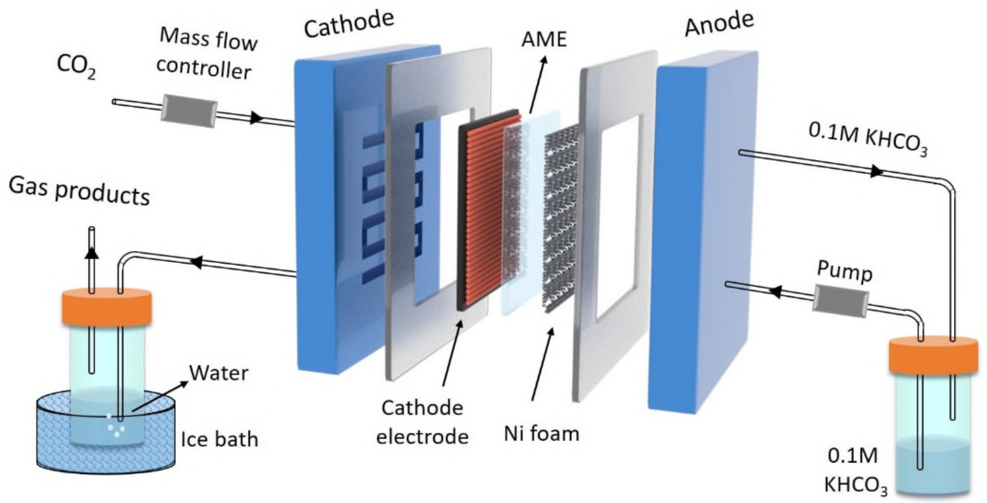

Supplementary Fig. 25 | Schematic for MEA. Total geometric area of flow field in the cathode is 4 cm^2, where the area of gas channel land is equal.

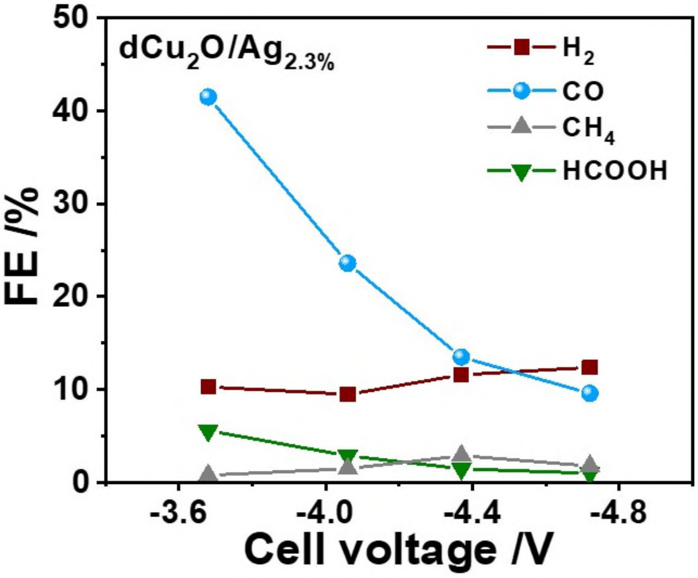

Supplementary Fig. 26 | FEs for C_1 and H_2 product in CO_2RR of $dCu_2O/Ag_{2.3\%}$ at cell voltage under MEA measurement.

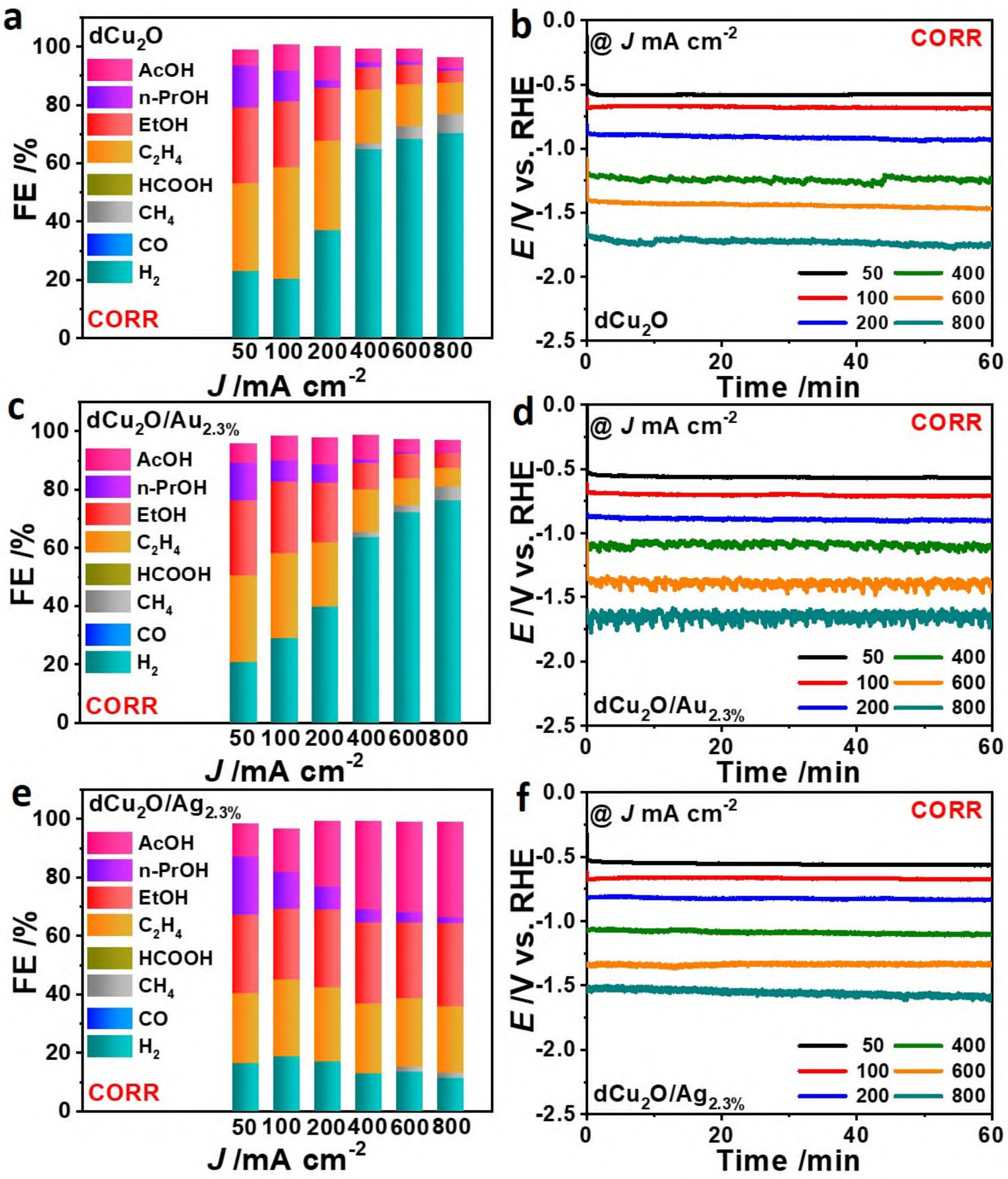

Supplementary Fig. 27 | (a, c, e) Product FEs and (b, d, f) Chronoamperometry curves for (a, b) dCu_2O, (c, d) $dCu_2O/Au_{2.3\%}$ and (e, f) $dCu_2O/Ag_{2.3\%}$ during CORR at applied current.

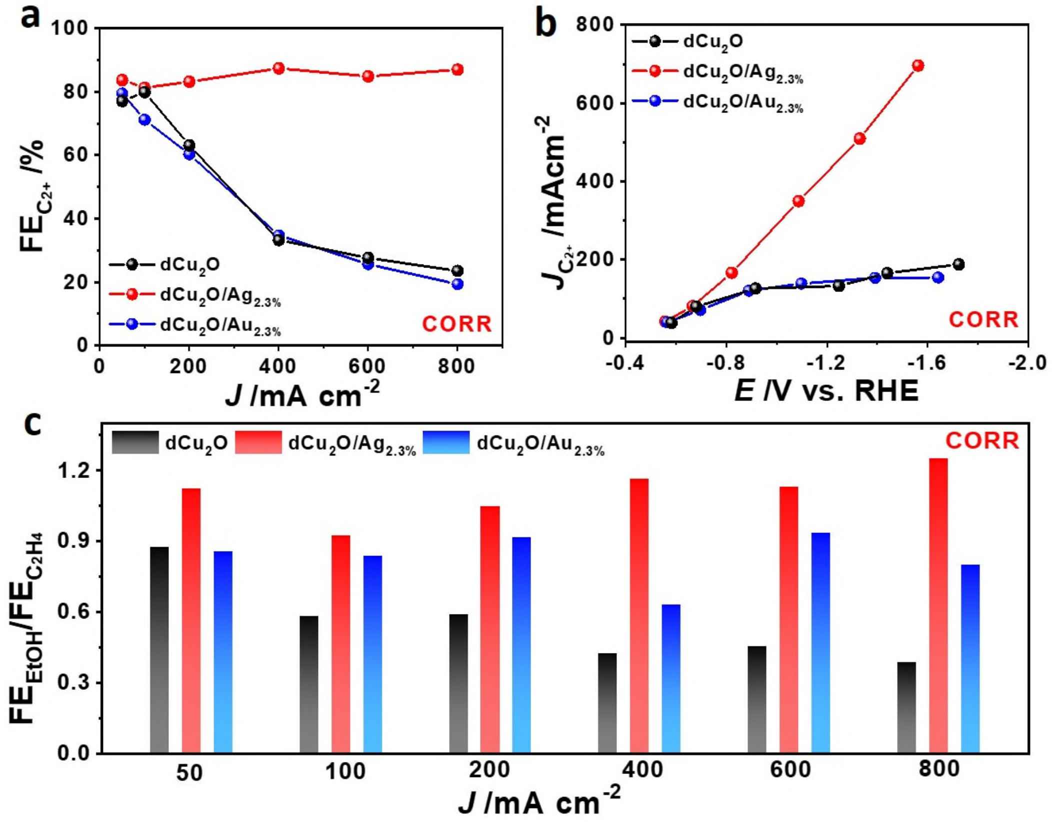

Supplementary Fig. 28 | CORR (a) FEs and (b) Partial current density for C_{2+} products on dCu_2O, $dCu_2O/Au_{2.3\%}$ and $dCu_2O/Ag_{2.3\%}$ under selected potential. (c) Ratio of FE_{EtOH} to FE_{C2H4} on dCu_2O, $dCu_2O/Ag_{2.3\%}$ and $dCu_2O/Au_{2.3\%}$ at current density.

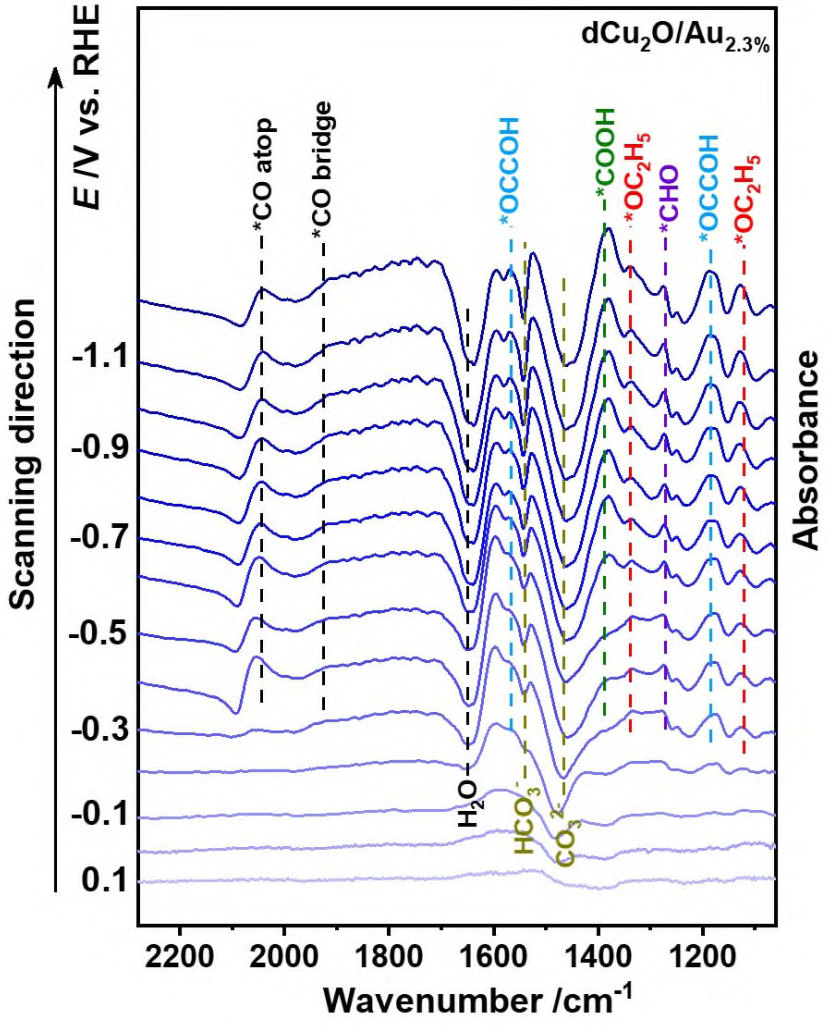

Supplementary Fig. 29 | *In situ* ATR–IRAS obtained during chronopotentiometry with a potential window of 0.2 to −1.2 V vs. RHE for $dCu_2O/Au_{2.3\%}$ in 1 M $KHCO_3$.

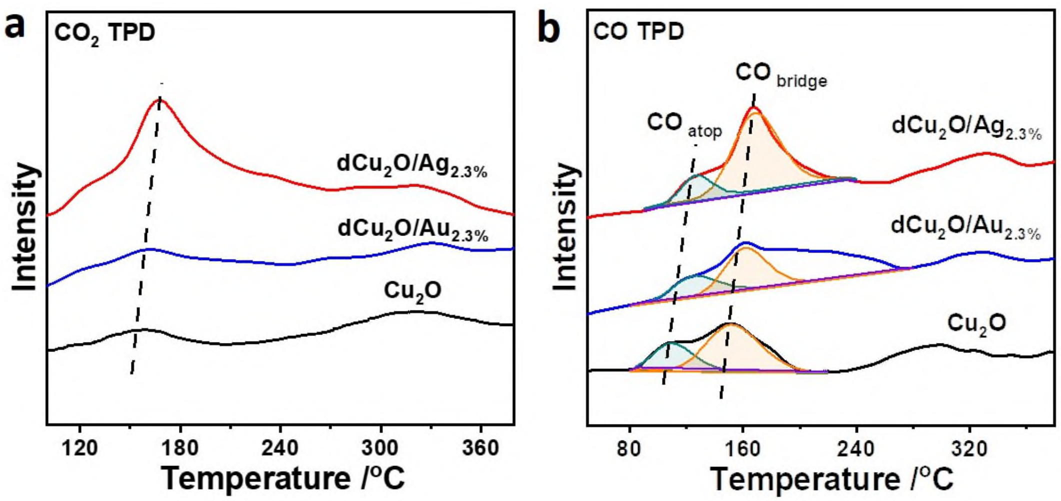

Supplementary Fig. 30 | (a) CO_2−TPD and (b) CO−TPD spectra for dCu_2O, $dCu_2O/Au_{2.3\%}$ and $dCu_2O/Ag_{2.3\%}$.

Note: Because of different test conditions, the $*CO_{bridge}$ peaks for dCu_2O and $dCu_2O/Au_{2.3\%}$ in CO-TPD are clear and different to ATR-IRAS data. However, the ratio $*CO_{bridge}/*CO_{atop}$ for $dCu_2O/Ag_{2.3\%}$ determined from the peak area remains greater than that for dCu_2O and $dCu_2O/Au_{2.3\%}$. This finding confirms that $dCu_2O/Ag_{2.3\%}$ favors forming $*CO_{bridge}$ compared with dCu_2O and $dCu_2O/Au_{2.3\%}$.

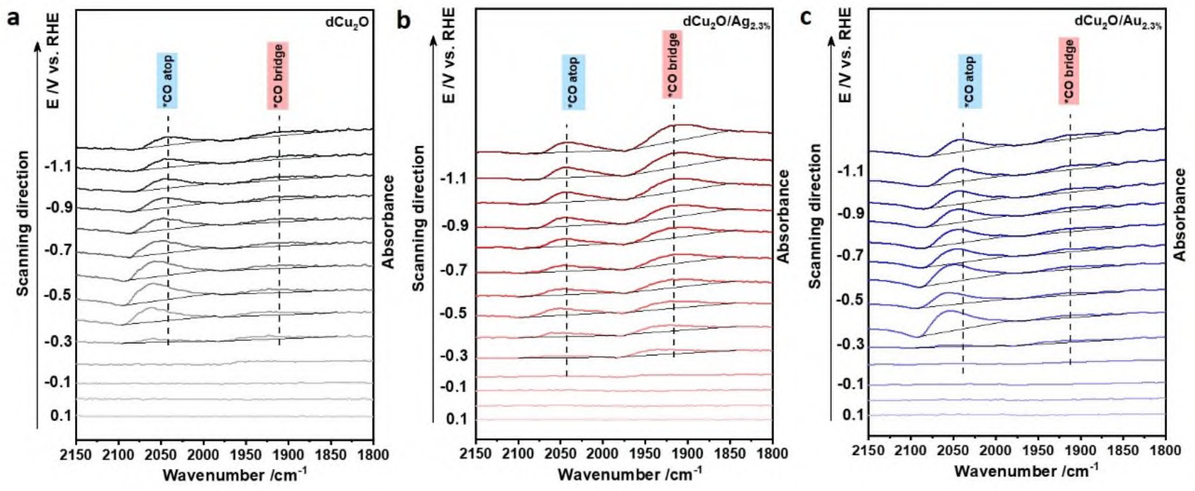

Supplementary Fig. 31 | Integration findings from *in situ* ATR–IRAS for $^{*}CO_{bridge}$ and $^{*}CO_{bridge}$ peaks around 2090 to 1980 and 1975 to 1844 cm^{-1} for (a) dCu_2O, (b) $dCu_2O/Ag_{2.3\%}$ and (c) $dCu_2O/Au_{2.3\%}$. Baseline construction used end-point weighted-mode with 5 % end point.

Supplementary Table 1. Cu K−edge EXAFS simulation parameters for selected catalysts.

Sample	Path	N	σ^2 (Å^2)	R (Å)	R-factor
Cu-foil	Cu-Cu	12	0.00877	2.54296	0.004
dCu_2O	Cu-Cu	11.4	0.00883	2.54315	0.005
	Cu-O	1.89	0.07992	1.88486	
$dCu_2O/Ag_{2.3\%}$	Cu-Cu	10.5	0.00884	2.54410	0.006
	Cu-O	1.75	0.04436	1.88073	
$dCu_2O/Au_{2.3\%}$	Cu-Cu	10.2	0.00878	2.54338	0.006
	Cu-O	1.70	0.03773	1.88722	

Supplementary Table 2. Electrocatalytic performance for CO_2 to EtOH product for recently reported catalysts.

Sample	Electrolyte	E (vs RHE)	$EE_{half\ cell}$ (EtOH, %)	FE (EtOH, %)	J_{total} (mA cm^{-2})	J_{EtOH} (mA cm^{-2})	Ref.
Cu_4Zn	0.1 M $KHCO_3$	-1.05	14.5	29.1	8.2	2.39	*ACS Catal.* **6**, 8239-8247 (2016)
Cu nanoparticle	1M KOH	-0.79	9.82	11	430	43.7	*J. Power Sources* **301**, 219-228 (2016)
Cu-DAT wire	1M KOH	-0.69	16.2	27.3	170	46.4	*ACS Catal.* **7**, 3313-3321 (2017)
Ag-Cu_2O-PB	0.2M KCl	-1.2	16.3	34.5	3	1.035	*ACS Catal.* **7**, 8594−8604 (2017)
Porous Cu	1M KOH	-0.67	9.96	16.6	653	108.4	*Adv. Mater.* **30**, 1803111-1803119 (2018)
CuAg-wire	1M KOH	-0.68	15.4	25	300	75	*J. Am. Chem. Soc.* **140**, 5791−5797 (2018)
CuS/Cu-V	1M KOH	-0.95	13.7	24.7	400	99	*Nat. Catal.* **1**, 421-428 (2018)
Abrupt Cu	1M KOH	-0.54	7.1	11	275	30.3	*Science* **360**, 783-787 (2018)
B-doped Cu	0.1 M $KHCO_3$	-1.1	13	27	70.4	19	*Nat. Chem.* **19**, 974-980 (2018)
$Ce(OH)_x$/Cu/PTFE	1M KOH	-0.7	25.2	42.6	300	128	*Nat. Commun.* **10**, 5814 (2019)
Cu/PTFE	1M KOH	-0.7	17.2	29.1	300	87.3	

Ag/Cu	1M KOH	-0.67	24.7	41.4	250	103.5	*J. Am. Chem. Soc.* **141**, 8584-8591 (2019)
Molecule-Cu	1M KOH	-0.8	23	41	300	124	*Nat. Catal.* **3**, 75-82 (2020)
N-C/Cu	1M KOH	-0.68	31	52	300	156	*Nat. Energy* **5**, 478-486 (2020)
F-Cu	1M KOH	-0.82	10.1	14	1600	224	*Nat. Catal.* **3**, 478-487 (2020)
$Cu_{0.8}Ag_{0.2}$	1M KOH	-0.78	18.3	32	330	105.6	*Cell Rep. Phys. Sci.* **1**, 100051 (2020)
Cu_3Ag_1	0.5 M $KHCO_3$	-0.95	20.6	39	70	17.2	*Adv. Energy Mater.* 2001987, (2020)
Cu_5Zn_8	0.1 M $KHCO_3$	-0.8	26.3	46.6	4	1.9	*Appl. Catal. B* **269**, 118800 (2020)
Cu/Cu_2O aerogels	0.1 m KCl	-1.1	20.3	41.2	81	33	*Adv. Funct. Mater.* **31**, 2102142 (2021)
Cu^+/hf-Cu	0.1 m KCl	-0.8	24.3	43	0.81	0.35	*Adv. Mater.* **34**, 2106028 (2021)
**dCu_2O/$Ag_{2.3\%}$**	**1M KOH**	**-0.87**	**22.3**	**40.8**	**800**	**326.4**	**This work**

Supplementary Table 3. Comparison of ECSA and mass normalized current density for EtOH for CO_2RR with independently reported catalysts.

**Sample**	**Mass loading (mg)**	**Geometric area (cm^2)**	**ECSA DLC (mF cm^{-2})**	**ECSA Pb UPD (cm^2)**	**J_{geo} (EtOH, mA cm^{-2})**	**J_{mass} (EtOH, (mA cm^{-2} mg^{-1})**	**$J_{ECSA\ DLC}$ (EtOH, mA mF^{-1})**	**$J_{ECSA\ Pb\ UPD}$ (EtOH, mA cm^{-2})**	**Ref.**
$Ce(OH)_x$/Cu/PTFE	~0.27	1	~3.07	/	128	~474.1	~41.7	/	*Nat. Commun.* **10**, 5814 (2019)
$Ag_{0.14}/Cu_{0.86}$	~0.27	1	1.68	/	103.5	~381.5	61.3	/	*J. Am. Chem. Soc.* **141**, 8584-8591 (2019)
F-Cu	~0.25	1	4.24	/	224	~896	52.8	/	*Nat. Catal.* **3**, 478-487 (2020)
Cu_{DS}	1	1	1.08	/	~75		69.4	/	*Joule*, **5** ,429-440 (2021)
NGQ/Cu-nr	~1	1	4.14	/	~115	~115	27.8	/	*Angew. Chem. Int. Ed.* **59**, 16459-16464 (2020)
Cu	/	4	/	20.3	~159	/	/	~7.83	*Angew. Chem. Int. Ed.* **60**, 14329-14333 (2021)
Cu-CuI	/	4	/	16.1	~233	/	/	~14.5	
Cu_2O HoMSs	/	/	/	~18.3	~187	/	/	~10.2	*Angew. Chem. Int. Ed.* **61**, e202113498 (2022)
$Cu_2P_2O_7$	4	4	/	17.8	~60	~15	/	3.37	*Angew. Chem. Int. Ed.* **61**, e202114238 (2022)
$dCu_2O/Ag_{2.3\%}$	**0.44**	**1**	**3.2**	**20.3**	**326**	**740.9**	**101.8**	**16.05**	**This work**

Supplementary Table 4. Comparison of detected intermediates and corresponding band positions of in situ ATR-FTIR for CO_2RR with independent studies.

**Band center (cm^{-1})**	**Band center (cm^{-1})**	**Assignment**	**Ref.**
**This work**	**Reported studies**		
~2044	~1951–2094	$*CO_{atop}$	*J. Am. Chem. Soc.* **142**, 2857–2867 (2020)
~1923	~1806–1930	$*CO_{bridge}$	
~1567 and ~1182	~1584 and ~1191	*OCCOH	*Angew. Chem. Int. Ed.* **56**, 3675-3678 (2017)
~1253	~1235	*HCO	
~1387	~1370	*COOH	*J. Am. Chem. Soc.* **139**, 15664-15667 (2017)
~1338	~1340	$*OC_2H_5$	*J. Phys. Chem. C* **123**, 5951–5963 (2019)
~1123	~1100	$*OC_2H_5$	*ACS Catal.* 4, 798-803 (2014)

Supplementary Table 5. The detailed synthetic parameters for Cu_2O/Ag and Cu_2O/Au NCs with different composition.

Sample	0.1M $Cu(NO_3)_2$ solution (mL)	1M NaOH solution (mL)	0.01M $AgNO_3$ solution (mL)	0.01M $HAuCl_4$ solution (mL)
Cu_2O	0.5	0.5	/	/
$Cu_2O/Ag_{1.2\%}$	0.5	0.5	0.05	/
$Cu_2O/Ag_{2.3\%}$	0.5	0.5	0.1	/
$Cu_2O/Ag_{3.0\%}$	0.5	0.5	0.15	/
$Cu_2O/Ag_{4.4\%}$	0.5	0.5	0.2	/
$Cu_2O/Au_{2.3\%}$	0.5	0.5	/	0.15
